# Enzyme replacement therapy for Anderson-Fabry disease: A complementary overview of a Cochrane publication through a linear regression and a pooled analysis of proportions from cohort studies

**DOI:** 10.1371/journal.pone.0173358

**Published:** 2017-03-15

**Authors:** Regina El Dib, Huda Gomaa, Alberto Ortiz, Juan Politei, Anil Kapoor, Fellype Barreto

**Affiliations:** 1 Institute of Science and Technology, Unesp - Univ Estadual Paulista, São José dos Campos, Brazil; 2 McMaster Institute of Urology, McMaster University, Hamilton, Canada; 3 Department of Pharmacy, Tanta Chest Hospital, Tanta, Egypt; 4 IIS-Fundacion Jimenez Diaz, Universidad Autonoma Madrid, Madrid, Spain; 5 Neurology Service, Dr Nestor Chamoles Laboratory of Neurochemistry, Buenos Aires, Argentina; 6 Department of Internal Medicine, Nephrology Service, Federal University of Paraná, Curitiba, Brazil; University of Florida College of Medicine, UNITED STATES

## Abstract

**Background:**

Anderson-Fabry disease (AFD) is an X-linked recessive inborn error of glycosphingolipid metabolism caused by a deficiency of alpha-galactosidase A. Renal failure, heart and cerebrovascular involvement reduce survival. A Cochrane review provided little evidence on the use of enzyme replacement therapy (ERT). We now complement this review through a linear regression and a pooled analysis of proportions from cohort studies.

**Objectives:**

To evaluate the efficacy and safety of ERT for AFD.

**Materials and methods:**

For the systematic review, a literature search was performed, from inception to March 2016, using Medline, EMBASE and LILACS. Inclusion criteria were cohort studies, patients with AFD on ERT or natural history, and at least one patient-important outcome (all-cause mortality, renal, cardiovascular or cerebrovascular events, and adverse events) reported. The pooled proportion and the confidence interval (CI) are shown for each outcome. Simple linear regressions for composite endpoints were performed.

**Results:**

77 cohort studies involving 15,305 participants proved eligible. The pooled proportions were as follows: a) for renal complications, agalsidase alfa 15.3% [95% CI 0.048, 0.303; I2 = 77.2%, p = 0.0005]; agalsidase beta 6% [95% CI 0.04, 0.07; I2 = not applicable]; and untreated patients 21.4% [95% CI 0.1522, 0.2835; I2 = 89.6%, p<0.0001]. Effect differences favored agalsidase beta compared to untreated patients; b) for cardiovascular complications, agalsidase alfa 28% [95% CI 0.07, 0.55; I2 = 96.7%, p<0.0001]; agalsidase beta 7% [95% CI 0.05, 0.08; I2 = not applicable]; and untreated patients 26.2% [95% CI 0.149, 0.394; I2 = 98.8%, p<0.0001]. Effect differences favored agalsidase beta compared to untreated patients; and c) for cerebrovascular complications, agalsidase alfa 11.1% [95% CI 0.058, 0.179; I2 = 70.5%, p = 0.0024]; agalsidase beta 3.5% [95% CI 0.024, 0.046; I2 = 0%, p = 0.4209]; and untreated patients 18.3% [95% CI 0.129, 0.245; I2 = 95% p < 0.0001]. Effect differences favored agalsidase beta over agalsidase alfa or untreated patients. A linear regression showed that Fabry patients receiving agalsidase alfa are more likely to have higher rates of composite endpoints compared to those receiving agalsidase beta.

**Conclusions:**

Agalsidase beta is associated to a significantly lower incidence of renal, cardiovascular and cerebrovascular events than no ERT, and to a significantly lower incidence of cerebrovascular events than agalsidase alfa. In view of these results, the use of agalsidase beta for preventing major organ complications related to AFD can be recommended.

## Introduction

Anderson-Fabry disease (AFD) is an X-linked recessive inborn error of glycosphingolipid metabolism caused by deficiency of alpha-galactosidase A (AGAL) which has an incidence estimated at 1 in 117,000 live births for males [1]; although a recent study suggest that the incidence may be much higher, particularly when non-classical phenotype is considered [2].

The complications of AFD include major renal, cardiac and cerebrovascular events due to progressive accumulation of a globotriaosylceramide (Gb3) in numerous cell types and of deacylated Gb3 (globotriaosylsphingosine, lyso-Gb3) in the circulation. Lyso-Gb3 promotes smooth muscle cell proliferation and podocyte injury in vitro [3,4].

Enzyme replacement therapy (ERT) is a specific medical treatment for AFD, and consists of intravenous infusion of the missing enzyme. There are two forms of recombinant human AGAL: agalsidase alfa (Replagal) and agalsidase beta (Fabrazyme). Agalsidase alfa produced in human fibrosarcoma cells; while agalsidase beta is produced by a Chinese Hamster Ovary cells. Both enzymes are approved in Europe and other countries, but the Federal Drug Agency (FDA) of the United States has approved only agalsidase beta [5,6]. ERT is infused biweekly. Surprisingly, the dose is five-fold different: 0.2 mg/kg body weight for agalsidase alfa and 1.0 mg/kg for agalsidase beta.

A proportion of people with AFD receiving ERT may develop anti-agalsidase antibodies; the occurrence of antibodies against agalsidase alfa and beta has been reported to be 55% and 83%, respectively [5,6]. The clinical efficacy seems not to be influenced by the antibody formation. Only in rare cases, IgE antibodies have been reported after infusion of agalsidase beta [5].

A Cochrane review [7] of nine randomized controlled trials (RCTs) addressing patient-important outcomes and including 351 patients reported that ERT is associated to significant improvement of endothelial deposits of globotriaosylceramide and of pain-related quality of life compared to placebo; however the effects of ERT on patient-important outcomes related to AFD was still unclear. In this regard, RCTs enrolled a limited number of patients and follow-up was relatively short, frequently just a few months.

The previous Cochrane review was limited since it did not include all studies in this rapidly evolving field and RCTs were mostly too short to be informative on key outcomes in this chronic rare disease that, for the kidney, has a mean natural history of four decades from birth to the need of renal replacement therapy. Regulatory authorities were well aware of the insufficient data obtained from RCTs and mandated the setting up of Registries that allowed the prospective collection of data on ERT efficacy and safety in larger number of patients followed for longer periods of time under routine clinical practice conditions. Although the ERT literature is dominated by data generated by these registries, until now no attempt at systematically reviewing and analyzing these data was available, thus depriving Fabry doctors and patients of a key source of information. Therefore, a complementary overview of a Cochrane publication through a linear regression and a pooled analysis of proportions from all cohort studies was undertaken.

## Material and methods

Our reporting adheres to the Preferred Reporting Items for Systematic Reviews and Meta-analyses (PRISMA) [8] (S1 Table) and Meta-analysis of Observational Studies in Epidemiology (MOOSE) Statements [9].

We performed a systematic review of clinical cohort studies with pooled analysis of proportions [10,11] and a linear regression of patients with AFD on ERT with either agalsidase alfa or beta.

### Eligibility criteria

- Study design: cohort studies (number of reported patients in each study greater than five). We excluded RCTs as this type of study was already evaluated in a Cochrane review [7].
- Participants: patients with genetically, enzymatically and/or biopsy-proven AFD regardless, age, gender, or disease severity.
- Interventions: agalsidase alfa, agalsidase beta, or untreated patients (i.e., natural history period, treatment-naïve patients).
- Patient-important outcomes:
  - All cause-mortality;
  - Renal events: end stage kidney disease needing dialysis; or kidney transplantation;
  - Cardiovascular events: myocardial infarction; needing cardiovascular devices; severe arrhythmia; or congestive heart failure;
  - Cerebrovascular events: stroke; or transitory ischemic attack.

Adverse events such as nasopharyngitis, rhinitis, nasal congestion, aggravation of allergic rhinitis with nasal discharge, dyspnea, cough, dizziness, flushing, pruritus, neuralgia, nausea, vomiting, diarrhea, abdominal pain, and arthralgia were also analyzed. We did not consider infusion reactions such as chills, fever, and headache as adverse events. We also did not consider serious adverse events as these could overlap with other clinical outcomes reported in the studies.

We excluded switching studies (for example, patients using agalsidase beta that was switched to agalsidase alfa) due to possible dose interaction effects. We also excluded studies that presented patients receiving either alfa or beta in the same set of analysis [12–16], and studies that did not specify the ERT used [17–19].

### Data source and searches

The Cochrane review with similar eligibility criteria retrieved studies up to September 2015 [7]. Using Medical Subject Headings (MeSH) based on the terms “Fabry Disease,” “agalsidase alfa,” and “agalsidase beta” (S1 Table) we replicated the search strategy of that review [7] for Medline, EMBASE, and LILACS from October 1, 2015 to March 1, 2016. We also included terms for “natural history”, “untreated patients,” “healthy control,” and “placebo” along with the terms for “Fabry Disease,” and we ran the search in the same electronic databases, without restriction of years (S2 Table). Furthermore, we reviewed reference lists of relevant review article [20] and primary studies. We did not impose any language restrictions. The search strategy was adapted for each database to achieve more sensitivity.

### Selection of studies

Reviewers independently screened all titles and abstracts identified by the literature search, obtained full-text articles of all potentially relevant studies, and evaluated them against the eligibility criteria. Reviewers resolved disagreement by discussion or, if necessary, with third party adjudication. We also considered studies reported only as conference abstracts.

### Data extraction

Reviewers independently extracted relevant data: study design; country; patients (number of patients, gender, mean age, children was defined as ≤ 18 years of age, phenotype); initiation of treatment; description of the intervention and control groups; outcomes event rates; and follow-up. We collected outcome data for the longest follow-up.

#### Multiple publications of the same study

If we found multiple publications from the same study and if supplementary reports included eligible outcomes not provided in the main report, we included complementary information from the multiple publications. However, we chose to extract total numbers of participants and events from the study that reported the largest sample size. Authors were contacted to clarify any issue related to multiple publications of the same study. Cohort studies with incomplete data were included only in the qualitative analysis.

### Statistical analysis

#### Proportional meta-analysis

We analyzed all outcomes as dichotomous variables with their respective confidence intervals (CI) of 95%. Since we expected that there were both clinical and methodological differences among the included studies, a random-effects model was used to perform the pooled analysis of proportions [21]. We only considered plotting into a meta-analysis studies that used the standard dose for agalsidase alfa (i.e., 0.2 mg/kg) and agalsidase beta (i.e., 1.0 mg/kg). We did not consider analyzing changes in globotriaosylceramide (Gb3, GL3) concentration in plasma, urine and tissue, or changes in lyso-Gb3 in plasma, neither pain in this review as these outcomes are continuous variables. The meta-analysis was performed with the StatsDirect software, version 2.8.0. (StatsDirect Ltd, Altrincham, Cheshire, UK).

Forest plots are presented to summarize data, and each horizontal line represents a study included in the meta-analysis. The length of the line corresponds to the 95% CI of the corresponding studies’ effect estimate. The effect estimate is marked with a solid black square. The size of the square represents the weight that the corresponding study exerts in the meta-analysis. The pooled estimate is marked with an unfilled diamond at the bottom of the forest plot. CIs of pooled estimates are displayed as a horizontal line through the diamond; this line might be contained within the diamond if the confidence interval is narrow. A statistically significant difference between two interventions required that their combined 95% CIs did not overlap [10,11].

#### Simple linear regressions and analysis of variance

Simple linear regressions for composite endpoints (death, renal or cardiac events, or stroke) were performed assuming a plausible best-case scenario analysis in which we considered the lowest event rate among those endpoints reported per included study. This was tested it using the general linear F-statistic. Simple linear regression was performed with the STATA software for Windows, version 14.1 (StataCorp. 2015. Stata Statistical Software: Release 14. College Station, TX: StataCorp LP).

Mean age and mean follow-up calculated in this study were based on the mean age and mean follow-up of each cohort study included in this review. Mean age was considered either at the beginning of ERT or the available age provided by the included studies for treated and untreated patients, respectively. We also performed analysis of variance (ANOVA) to compare mean number of male and mean age among the three studied groups (alfa, beta, and untreated). This analysis was performed with SAS/STAT^®^ software for Windows, version 9.3 (SAS Institute Inc. Trademarks).

### Statistical heterogeneity and sensitivity analysis

We planned to perform sensitivity analysis by gender (i.e. male versus female), age (i.e. adults versus children), follow-up periods (< 5 years versus ≥ 5 years), and AFD phenotype (i.e. classical versus non-classical); however we were only able to perform sensitivity analysis for age and different follow-up periods because there were an insufficient number of studies to allow this analysis for the other two variables. We considered inconsistent results between the primary and sensitivity analysis when the difference between the proportions was more than 3%.

We also planned to assess publication bias through visual inspection of funnel plots for each outcome in which we identified 15 or more eligible studies. We used I^2^ statistic to test for heterogeneity, and significance was assumed when I^2^ was > than 50% with a P < 0.1.

## Results

### Study selection

**Fig 1:**
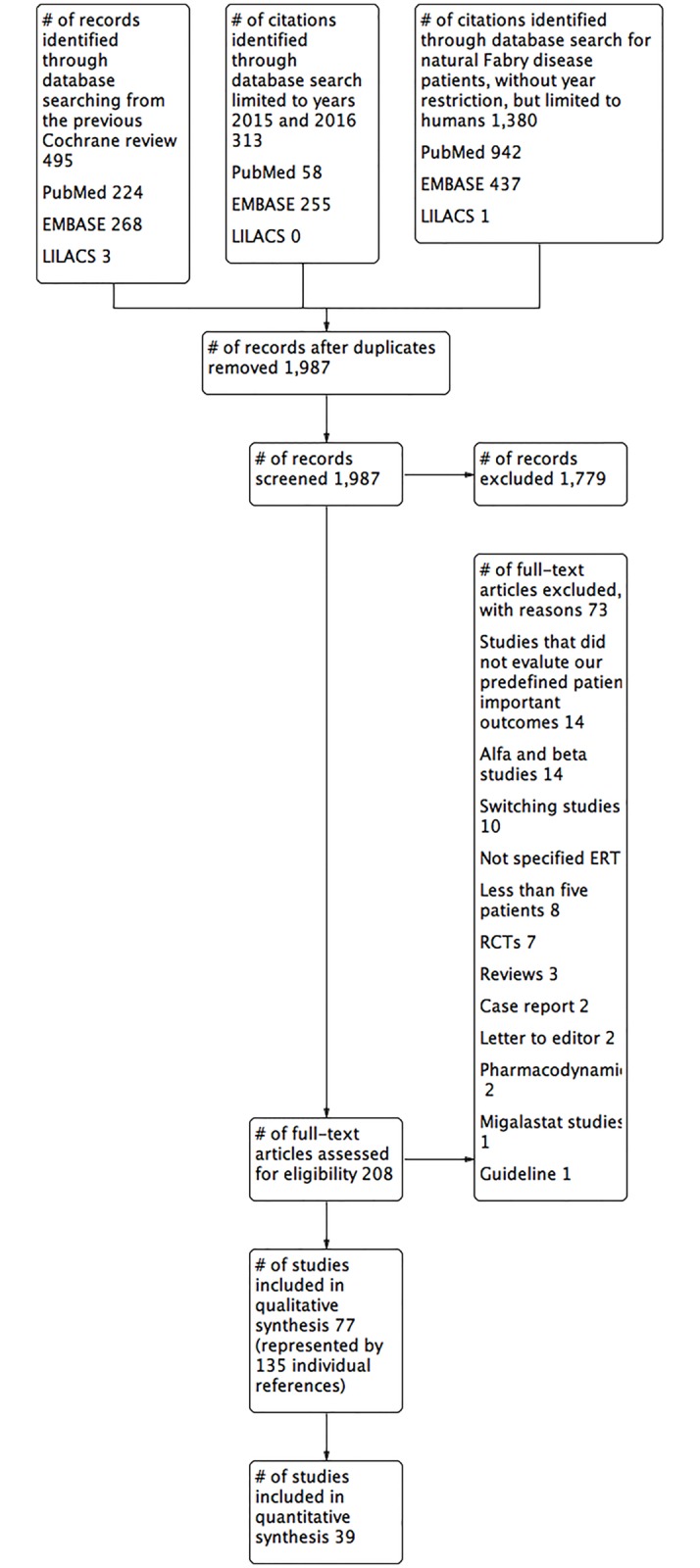
Flowchart of the review.

Fig 1 presents the process of identifying eligible studies, including publications in the Cochrane systematic review [7], and citations identified by searching in electronic databases. We identified a total of 1,987 citations, after duplicates were removed. Based on title and abstract screening, we assessed 208 full-texts of which we included 77 cohort studies (represented by 135 individual references) involving 15,305 participants. The majority of the included studies (79.2%; n = 61) were available as full-text articles and 20.7% (n = 16) were only abstracts reports.

We contacted the authors of all the included studies, but only nine [22–34] responded whether there was an overlap of patients in their multiple publications (S3 Table).

### Study characteristics

Table 1 describes study characteristics related to country, number of participants, mean age, gender, initiation of treatment, and mean follow-up for different ERT regimens. Thirty-four studies [25–27,32,33,35–63] were conducted largely in Europe, 16 were international studies [22–24, 64–76], 10 in the USA [28–30,77–83], nine in Asia [84–92], six in Central and South America [93–98], and two in Canada [31,34]. Cohort studies sample size ranged from six [60,93] to 2,869 [68]. Typical participants were males in their 30s and 40s. There was no statistically significance difference related to mean age among the three studied groups (p = 0.10); however there was a statistically significance difference in the percentage of males between the agalsidase beta (74.7%) and untreated groups (49.1%) (p = 0.01). Studies followed participants from five months [85] to 24.25 years [42] with a mean follow-up of 3.8 years.

**Table 1:** Characteristics of patients undergoing ERT: Comparison among different regimens for AFD patients.

	Total	Alfa	Beta	Untreated
**Total cohort studies**	77	29	31	20^$^
**Total number of patients**	15,305	2,840	3,598	8,867
Mean percentage of males*^£^	66.4	65.9	74.7	49.1
Mean age*^€^ **(years)**	35.1	34.4	33.1	41.2
**Total number of children**	277	194	50	33
**Phenotype** (# of studies):				
Classic	7	2	3	3
Non-classic	0	0	0	0
Cassic and non-classic	4	0	1	2
Not reported	66	27	27	15^$^
Total number of classic patients	1,407	26	1,060	321
**Initiation of treatment** (# of studies):				
≤ 25 year-old	7	4	3	NA
> 25 year-old	13	8	5	NA
≤ 25 and > 25 year-old	11	4	7	NA
Not reported	29	13	16	NA
**Country** (# of studies):				
Europe	34	12	15	7^$^
International	16	7	5	6^$^
USA	10	4	2	4
Asian	9	2	6	1
Central and South America	6	3	2	1
Canada	2	1	1	1
**Mean follow-up (years)**	3.8	3.3	3.9	4.5

NA: not applicable.

*In the whole population.

^$^Three studies [23,62,66] also evaluated agalsidase beta.

^£^There was a statistically significance difference between agalsidase beta and untreated groups (p = 0.0192).

^€^There was no statistically significance difference among the three studied groups (p = 0.1050).

There were more studies evaluating agalsidase beta (41.2%, n = 31) with a total of 3,598 patients) followed by agalsidase alfa (37.6%, n = 29 with a total of 2,840 patients), and untreated patients, including natural history period of AFD patients and healthy volunteers (25.9%, n = 20 with a total of 8,865 patients). Only 1.8% (n = 277) of the total sample size evaluated was children (Table 1).

Four (12.9%) [39,51,80,93] of the 31 agalsidade beta studies specified the phenotype of AFD, two [51,93] as classic, and further two [39,80] as classic and non-classic patients. Only two (6.9%) [36,41] of the 29 agalsidase alfa studies specified the disease as classic AFD (Table 1).

S4 Table describes study characteristics related to description of the intervention, inclusion and exclusion criteria. The majority of the included studies reported on a confirmed diagnosis of AFD by detection of mutations in the GLA, gene, or by alpha-galactosidase A enzymatic assay.

### Outcomes

#### All-cause mortality

**Fig 2:**
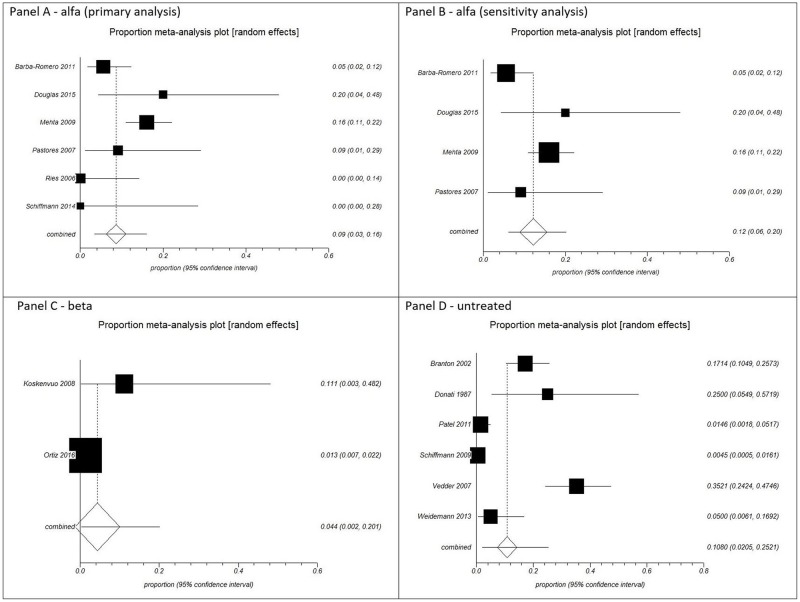
Pooled analysis of proportions from cohort studies for all-cause mortality. Panel A: agalsidase alfa (primary analysis). Panel B: agalsidase alfa (sensitivity analysis excluding children). Panel C: agalsidase beta. Panel D: untreated patients.

The pooled proportions for all-cause mortality were: agalsidase alfa (primary analysis) from six cohort studies [28, 37,43,67,70,83] with a total of 344 patients, 9% [95% CI 0.03, 0.16; I^2^ = 68.9%, p = 0.0066]; agalsidase alfa (sensitivity analysis excluding children) from four cohort studies [37,43,67,83] with a total of 309 patients, 12% [95% CI 0.06, 0.20; I^2^ = 62.2%, p = 0.0475]; agalsidase beta from two cohort studies [53,80] with a total of 1,053 patients, 4.4% [95% CI 0.002, 0.201; I^2^ = not applicable]; and untreated patients from six cohort studies [29,33,42,62,68,77] with a total of 812 patients, 10.8% [94.5% CI 0.0205, 0.2521; I^2^ = 95.6%, p < 0.0001] (Fig 2). There was significance regarding heterogeneity in all analyses, except for agalsidase beta as the I^2^ was not applicable. A plausible sensitivity analysis excluding children for agalsidase alfa yielded results that were inconsistent with the primary analysis showing almost a quarter more deaths (Fig 2).

Although the rate of all-cause mortality in the primary analysis was higher in the untreated patients (10.8%), followed by agalsidase alfa (9%), and agalsidase beta (4.4%), there was no significant difference between the groups, as their CIs overlapped (Fig 2).

#### Renal complications

The pooled proportions for renal complications were: agalsidase alfa (primary analysis) from six cohort studies [27,37,43,58,70,98] with a total of 168 patients, 15.3% [95% CI 0.048, 0.303; I^2^ = 77.2%, p = 0.0005]; agalsidase alfa (sensitivity analysis excluding children) from five cohort studies [27,37,43,58,98] with a total of 152 patients, 16.8% [95% CI 0.041, 0.356; I^2^ = 81.4%, p = 0.0002]; agalsidase beta from one cohort [80] with a total of 1,044 patients 6% [95% CI 0.04, 0.07; I^2^ = not applicable]; and untreated patients from 11 cohort studies [29,33,42,47,56,62,65,68,77,89,97] with a total of 1,698 patients, 21.4% [95% CI 0.1522, 0.2835; I^2^ = 89.6%, p < 0.0001] (S1 Fig). There was significance regarding heterogeneity in all analyses, except for agalsidase beta as the I^2^ was not applicable. A plausible sensitivity analysis excluding children for the agalsidase alfa yielded results that were consistent with the primary analysis (S1 Fig).

**Fig 3:**
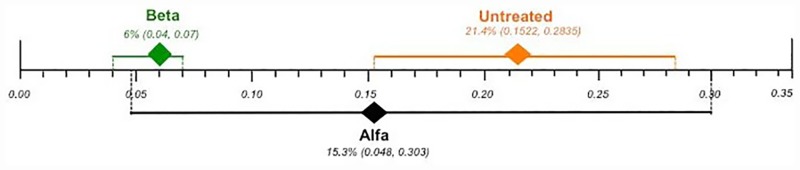
Comparison of the plotted proportional meta-analysis, according to ERT regimens, for renal complications. Effect differences were seen due to the non-overlap of the 95% confidence intervals favoring the use of agalsidase beta compared to untreated patients, as their CIs did not overlap. There was no statistically significance difference between agalsidase alfa and both untreated patients and agalsidase beta, as their CIs overlapped.

Effect differences were observed since the 95% CIs did not overlap, favoring the use of agalsidase beta over untreated patients. There was no statistically significance difference between agalsidase alfa and untreated patients or agalsidase beta, as their CIs overlapped (Fig 3).

#### Cardiovascular complications

The pooled proportions for cardiovascular complications were: agalsidase alfa (primary analysis) from four cohort studies [43,54,67,69] with a total of 524 patients, 28% [95% CI 0.07, 0.55; I^2^ = 96.7%, p < 0.0001]; agalsidase alfa (sensitivity analysis excluding children) from three cohort studies [43,54,67] with a total of 426 patients, 35% [95% CI 0.11, 0.65; I^2^ = 95.1%, p < 0.0001]; agalsidase beta (primary analysis) from three cohort studies [59,75,80] with a total of 1,069 patients, 7% [95% CI 0.05, 0.08; I^2^ = not applicable]; agalsidase beta (sensitivity analysis excluding children) from two cohort studies [59,80] with a total of 1,053 patients, 7% [95% CI 0.05, 0.08; I^2^ = not applicable]; and untreated patients from 14 cohort studies [31,33,42,47,54,56,60,65,68,72,76,80,89,97] with a total of 5,854 patients, 26.2% [95% CI 0.149, 0.394; I^2^ = 98.8%, p < 0.0001] (S2 Fig). There was significance regarding heterogeneity in all analyses, except for agalsidase beta, as the I^2^ was not applicable. A plausible sensitivity analysis excluding children for the agalsidase alfa yielded results that were inconsistent with the primary analysis showing a difference of 7% more deaths when excluding studies that evaluated children. However, a plausible sensitivity analysis excluding children for the agalsidase beta yielded results that were consistent with the primary analysis (S2 Fig).

**Fig 4:**
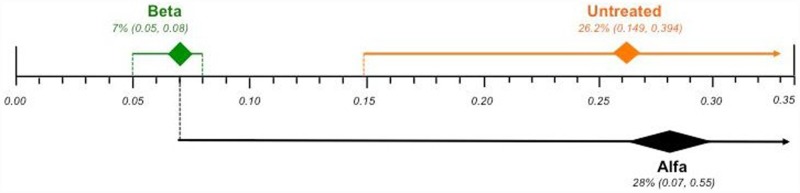
Comparison of the plotted proportional meta-analysis, according to ERT regimens, for cardiovascular complications. Effect differences were seen due to the non-overlap of the 95% confidence intervals favoring the use of agalsidase beta compared to untreated patients, as their CIs did not overlap. There was no statistically significance difference between agalsidase alfa and both untreated patients and agalsidase beta, as their CIs overlapped.

Effect differences were observed, since the 95% CI did not overlap, favoring the use of agalsidase beta over untreated patients. However, there was no statistically significance difference between agalsidase alfa and both untreated patients and agalsidase beta, as their CIs overlapped (Fig 4).

#### Cerebrovascular complications

The pooled proportions for cerebrovascular complications were: agalsidase alfa (primary analysis) from seven cohort studies [37,43,63,67,69,70,98] with a total of 461 patients, 11.1% [95% CI 0.058, 0.179; I^2^ = 70.5%, p = 0.0024]; agalsidase alfa (sensitivity analysis excluding children) from five cohort studies [37,43,63,67,98] with a total of 339 patients, 10.5% [95% CI 0.043, 0.190; I^2^ = 71.2%, p = 0.0077]; agalsidase beta from three cohort studies [53,59,80] with a total of 1,062 patients, 3.5% [95% CI 0.024, 0.046; I^2^ = 0%, p = 0.4209]; and untreated patients from 15 cohort studies [29,31,33,42,47,56,60,62,65,68,72,78,81,89,97] with a total of 5,544 patients, 17.8% [95% CI 0.123, 0.240; I^2^ = 95.2% p < 0.0001]. There was significance regarding heterogeneity in all analyses, except for agalsidase beta as the I^2^ was 0%. A plausible sensitivity analysis excluding children for agalsidase alfa yielded results that were consistent with the primary analysis (S3 Fig).

**Fig 5:**
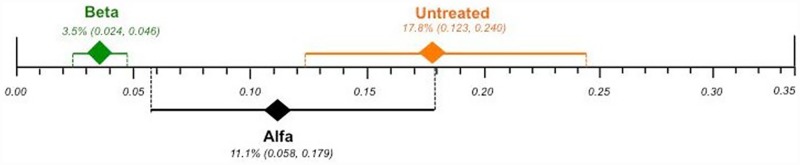
Comparison of the plotted proportional meta-analysis, according to ERT regimens, for cerebrovascular complications. Effect differences were seen due to the non-overlap of the 95% confidence intervals favoring the use of agalsidase beta compared to both untreated patients and agalsidase alfa, as their CIs did not overlap. There was no statistically significance difference between agalsidase alfa and untreated patients, as their CIs overlapped.

Effect differences were observed, since the 95% CI did not overlap, favoring agalsidase beta over both agalsidase alfa and untreated patients. There was no statistically significance difference between agalsidase alfa and untreated patients, as their CIs overlapped (Fig 5).

#### Adverse events

The pooled proportions for adverse events were: agalsidase alfa (primary analysis) from nine cohort studies [28,41,45,67,69,70,83,95,98] with a total of 482 patients, 31.3% [95% CI 0.149, 0.507; I^2^ = 93.4%, p <0.0001]; agalsidase alfa (sensitivity analysis excluding children) from six cohort studies [41,45,67,83,95,98] with a total of 349 patients, 27% [95% CI 0.088, 0.505; I^2^ = 93.8%, p < 0.0001]; agalsidase beta (primary analysis) from five cohort studies [39,59,75,79,80] with a total of 1,089 patients, 34.1% [95% CI 0.071, 0.688; I^2^ = 93.3%, p < 0.0001]; agalsidase beta (sensitivity analysis excluding children) from four cohort studies [39,59,79,80] with a total of 1,073 patients, 19.5% [95% CI 0.055, 0.393; I^2^ = 74.8%, p = 0.0077]; and untreated patients from one cohort [47] with a total of 11 patients, 37% [95% CI 0.13, 0.65; I^2^ = not applicable]. There was significant heterogeneity in all analyses, except in the untreated group, as the I^2^ was not applicable. A plausible sensitivity analysis excluding children for both agalsidase alfa and beta yielded results that were inconsistent with the primary analysis (S4 Fig).

There was no statistically significance difference between agalsidase alfa, agalsidase beta and untreated groups related to adverse effects rates, as their CIs overlapped (S4 Fig).

A second sensitivity analysis from the same nine cohort studies [28,41,45,67,69,70,83,95,98] on agalsidase alfa, examined whether different follows up (≥ 5 years versus < 5 years) differed substantially. There were no substantial differences in the point estimates between ≥ 5 years follow-up with three included studies [28,67,95] (n = 286 patients) (37.0% [95% CI 0.08, 0.74; I^2^ = 96.6%, p <0.0001]) and < 5 years follow-up with six included studies [41,45,69,70,83,98] (n = 196 patients) (28.6% [95% CI 0.082, 0.554; I^2^ = 92.2%, p <0.0001]) (S5 Fig). Furthermore, there was also no statistically significany difference between the sensitivity analyses for different follow-up periods for agalsidase alfa compared to agalsidase beta and untreated groups, as their CIs overlapped (S5 Fig).

#### Composite endpoints

**Fig 6:**
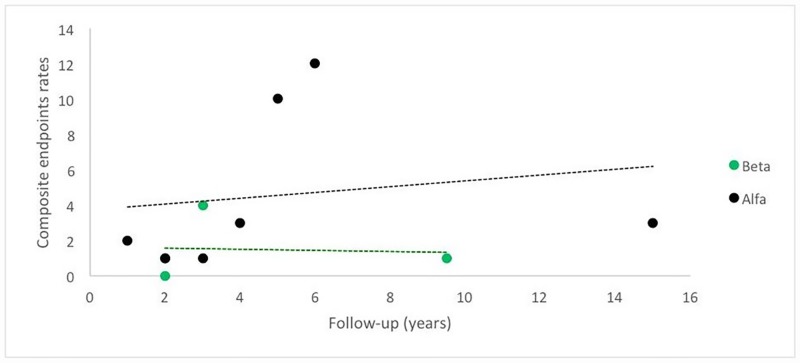
Progression of composite endpoints, according to ERT regimens throughout the years.

Data from seven [30,43,58,63,67,83,98] and four [23,53,59,62] studies included in the alfa and beta groups, respectively, with a follow-up from one year to 15 years in agalsidase alfa, and a follow-up from two years to 9.5 years in agalsidase beta, assessed by simple linear regression showed that Fabry patients receiving agalsidase alfa are more likely to have higher rates of composite endpoints (death, renal, cardiovascular or cerebrovascular events) compared to those receiving agalsidase beta throughout the years; however there was no statistically significant difference (p = 0.5878) (Fig 6).

## Discussion

### Main findings

AFD is a rare disease with a long natural history. For example, it takes approximately four decades since birth, when glycolipid deposits are already present in tissue, to the development of end-stage renal disease [81]. This poses several problems for evidence-based medicine. Being a rare disease, it is very difficult to recruit enough patients to develop RCTs with hard end-points as primary outcome measures. Furthermore, the long natural history implies a very long follow-up, which may turn RCTs unfeasible, or, alternatively, enroll patients in RCTs when the disease is already advanced, which severely limits the ability to demonstrate benefit since advanced tissue injury usually progresses despite removal of the original cause. On top of this, any placebo-controlled clinical trial is unethical given the availability of therapy. Thus, further acquisition of knowledge needs to rely on observational studies, mainly from the two registries (Fabry Outcome Survey (FOS) and Fabry Registry) mandated by regulatory agencies for this purposes. We have developed the first systematic approach to the analysis of data generated by the registries and other cohort studies.

We have now applied new methodology [10,11] to evaluate the efficacy and safety of ERT for AFD based on available observational studies in order to complement available information from RCTs and meta-analysis of RCTs. The main finding is that the data are consistent with the efficacy of ERT in preventing hard outcomes, including renal, cardiac and cerebrovascular complications. However, the most striking observation was that the benefit was limited to agalsidase beta. While a higher number of patients were treated with agalsidase beta in the included studies, and this may have favored the observation of statistically significant differences over the control groups, a trend was observed for better outcomes in all event categories for agalsidase beta over alfa that was significant for cerebrovascular events. Indeed, there is a biological plausibility for the observation. Thus, agalsidase alfa is prescribed at 5-fold lower dose that agalsidase beta, resulting in 5- to 10-fold lower intracellular agalsidase activity [99]. A case series suggest that the lower dose may not adequately clear glycolipids from certain cells such as podocytes [100]. Indeed, preliminary data reported in abstract form from a head-to-head comparison between agalsidase alfa and agalsidase beta have so far disclosed a non-significant nearly 2-fold higher incidence of severe clinical events per patient on agalsidase alfa than on agalsidase beta [101].

### Strengths and limitations

Strengths of our study include a comprehensive search; assessment of eligibility, a data abstraction which was independent and in duplicate.

The primary limitation of our study is related to the rare disease nature of AFDs. Thus, the population available to study was limited and in some studies long-term follow-up was not available.

Another limitation is that our analysis demonstratesd a significant heterogeneity in the clinical outcome of all studied groups (i.e. alfa, beta, and untreated patients). Explanations for this heterogeneity could be both clinical and methodological diversities. The studies differed considerably in their patient selection (i.e. phenotype, or more frequently, studies that did not specify the type of Fabry), baseline disease severity (e.g. different co-morbidities associated), and ERT regimens (i.e. alfa, beta).

Furthermore, out of the 77 cohort studies we were only able to include data in the meta-analysis from 39 of them (50.6%). The majority of the studies were difficult to decipher, and they did not provide all patient-important outcomes. We were also struggling with the fact that there were multiple publications of the same set of patients, and we attempted to avoid overlapping of same patients in the meta-analysis. Methodological studies have demonstrated that failure to account for overlapping patients can increase type I error when combining results from multiple studies in the same meta-analysis [102]. To deal with this issue we contacted the authors of the included studies to request information on the overlap of patients in their publications (S3 Table). Unfortunately, only 11.7% of the authors replied addressing our concern. Furthermore, since major centers are enrolling their patients in either the FOS or the Fabry Registry, where data is anonymized, even authors may not know whether specific patients are also included in Registry-based papers.

A further limitation was the insufficient number of studies that did not allow completing statistical analysis initially planned. We were not able to assess publication bias because there were less than 15 eligible studies addressing the same outcome in a meta-analysis. Subgroup analyses according to gender (male versus female), phenotype (classical versus non-classical) or disease severity at start of therapy was not possible since minimal criteria were not met (i.e. at least six studies available, with at least three in each sub-group).

Another limitation is that individual patient data were not available. This precludes optimal assessment of the impact of length of follow-up, which would require a time-to-event analysis. A sensitivity analysis pooling the short-term studies (i.e. < 5 years) separately from long-term studies (i.e. ≥ 5 years) was only possible for adverse events and no difference was found in the proportion of events between both analyses.

One could argue that these limitations make the pooling of results inadvisable. However, the analyses suggest a possible superior efficacy of agalsidase beta over untreated patients for all events; and over alfa on the risk of cerebrovascular events. To the best of our knowledge, this is the first study to clearly demonstrate the benefits of using ERT, an observation that should be taken into account in health policy debates regarding Fabry disease. In this regard, the results are not surprising. Rather, they lend further support to results from small RCTs. Agalsidase beta was superior to placebo in the only available RCT (n = 82) with primary end-point of severe clinical events [103]. However, the difference was only significant in a per protocol analysis after a pre-specified adjustment for baseline proteinuria. No such RCT is available for agalsidase alfa.

The final weakness to be acknowledged is that given the unavailability of information in the analyzed studies, we were unable to clearly separate pre- and post-2001 natural history cohorts or to analyze only classical Fabry patients. However, this weakness may have biased the results against finding the differences that were observed. Thus, the largest agalsidase beta cohort consisted of >1,000 classic patients. By comparing this large classical cohort with untreated patients in whom phenotype was not specified (and may include milder late-onset phenotypes) or who may remain untreated post-2001 (potentially because of a milder phenotype), we may have hedged the odds against observing a difference.

### Relation to prior work

The previous Cochrane review [7] addressing only RCTs studies concluded that ERT improved endothelial deposits of GL3 and pain-related quality of life when compared to placebo. However, there was no evidence of benefit on major clinical outcomes neither on the superiority of one of the current available ERT. However, the absence of the highest level of evidence for an intervention does not necessarily mean that it is ineffective or clinically useless. Thus, misinterpreting the Cochrane review might mislead therapeutic decisions on AFD.

A Cochrane review’s conclusions are based strictly on the included studies, that should be RCTs. RCTs are particularly scarce and underpowered in the setting of rare diseases. In this context, a study evaluating the robustness of overall conclusions from 1,016 Cochrane systematic reviews of RCTs found that 96% of the analyzed reviews recommended further research [104]. Therefore, most authors of these systematic reviews concluded that there was insufficient evidence to answer the clinical questions on therapeutic strategies [104]. This is of little help to practicing physicians that have to make therapeutic decisions on a daily basis.

Meta-analysis optimizes the results of a systematic review. However, heterogeneity and/or insufficient number of studies addressing the same clinical question limit the ability to perform meta-analysis. Indeed, for most of the 1016 systematic reviews, it was only possible to identify two clinical trials that satisfied the inclusion criteria [104]. The same team of authors reanalyzed in 2011 a random sample of Cochrane systematic reviews and found similar percentages in the analysis of 1,128 systematic reviews [105].

Furthermore, the term "very low quality evidence" means that there was not enough or well-conducted included studies to determine whether the treatment under study is more effective compared to the control group and; again does not necessarily mean that it is ineffective or clinically useless. Systematic reviews use rigorous methods to identify, critically appraise and synthesize relevant research studies [106]. Nonetheless, as verified by some studies [104,105] they lack sufficient numbers of clinical trials. Moreover, readers of systematic reviews usually, but erroneously, conclude "absence of effect" or "absence of differences between treatments" instead of "there is insufficient evidence either to support or to refute" [107].

A Cochrane review [108] evaluating the efficacy and safety of ERT and substrate reduction therapy for treating Gaucher disease identified the low sample size of RCTs as a real challenge for the analysis of rare diseases.

## Conclusions

Agalsidase beta is associated to a significantly lower incidence of renal, cardiovascular and cerebrovascular events than no ERT, and to a significantly lower incidence of cerebrovascular events than agalsidase alfa. The rates of adverse events between agalsidase alfa and beta did not differ statistically. In view of these results, the use of agalsidase beta for preventing major organ complications related to AFD can be recommended. This study also highlights the need to address the suboptimal reporting of cohort studies through the development of a guidance document focused on reporting studies on rare diseases.

## Supporting information

S1 Table PRISMA checklist. (DOC)

S2 Table Search strategy. (DOCX)

S3 Table Information about contact with authors of the included studies. (DOCX)

S4 Table Study characteristics related to inclusion and exclusion criteria per included study. (DOCX)

S1 Fig Pooled analysis of proportions from cohort studies for renal complications. Panel A: agalsidase alfa (primary analysis). Panel B: agalsidase alfa (sensitivity analysis excluding children). Panel C: agalsidase beta. Panel D: untreated patients.
(TIF)

S2 Fig Pooled analysis of proportions from cohort studies for cardiovascular complications. Panel A: agalsidase alfa (primary analysis). Panel B: agalsidase alfa (sensitivity analysis excluding children). Panel C: agalsidase beta (primary analysis). Panel D: agalsidase beta (sensitivity analysis excluding children). Panel E: untreated patients.
(TIF)

S3 Fig Pooled analysis of proportions from cohort studies for cerebrovascular complications. Panel A: agalsidase alfa (primary analysis). Panel B: agalsidase alfa (sensitivity analysis excluding children). Panel C: agalsidase beta. Panel D: untreated patients.
(TIF)

S4 Fig Pooled analysis of proportions from cohort studies for adverse events. Panel A: agalsidase alfa (primary analysis). Panel B: agalsidase alfa (sensitivity analysis excluding children). Panel C: agalsidase beta (primary analysis). Panel D: agalsidase beta (sensitivity analysis excluding children). Panel E: untreated patients.
(TIF)

S5 Fig Sensitivity analysis comparing different follow-up periods in agalsidase alfa in primary analysis. Panel A: ≥ 5 years. Panel B: < 5 years.
(TIF)

## Abbreviations

AFD: Anderson-Fabry disease
AGAL: alpha-galactosidase A
CHO: Chinese hamster ovary
CI: confidence interval
ERT: enzyme replacement therapy
Gb3: globotriaosylsphingosine
MOOSE: Meta-analysis of Observational Studies in Epidemiology
PRISMA: Preferred Reporting Items for Systematic Reviews and Meta-analyses
RCTs: randomized controlled trials

## References

[1] Meikle PJ, Hopwood JJ, Clague AE, Carey WF. Prevalence of lysosomal storage disorders. JAMA 1999;281:249–54. 9918480 10.1001/jama.281.3.249

[2] Spada M, Pagliardini S, Yasuda M, Tukel T, Thiagarajan G, Sakuraba H, et al High incidence of later-onset Fabry disease revealed by newborn screening. American Journal of Human Genetics 2006;79:31–40. 10.1086/504601 16773563 PMC1474133

[3] Aerts JM, Groener JE, Kuiper S, Donker-Koopman WE, Strijland A, Ottenhoff R, et al Elevated globotriaosylsphingosine is a hallmark of Fabry disease. Proceedings of the National Academy of Sciences of the United States of America 2008;105:2812–7. 10.1073/pnas.0712309105 18287059 PMC2268542

[4] Sanchez-Niño MD, Carpio D, Sanz AB, Ruiz-Ortega M, Mezzano S, Ortiz A. Lyso-Gb3 activates Notch1 in human podocytes. Hum Mol Genet. 2015;24:5720–32. 10.1093/hmg/ddv291 26206887

[5] Eng CM, Guffon N, Wilcox WR, Germain DP, Lee P, Waldek S, International Collaborative Fabry Disease Study Group, et al Safety and efficacy of recombinant human alpha-galactosidase A—replacement therapy in Fabry’s disease. New England Journal of Medicine 2001;345: 9–16. 10.1056/NEJM200107053450102 11439963

[6] Schiffmann R, Kopp JB, Austin HA, Sabnis S, Moore DF, Weibel T, et al Enzyme replacement therapy in Fabry disease: a randomized controlled trial. JAMA 2001;285:2743–9. 11386930 10.1001/jama.285.21.2743

[7] El Dib R Gomaa H, Carvalho RP, Camargo SE, Bazan R, Barretti P, Barreto FC. Enzyme replacement therapy for Anderson-Fabry disease. Cochrane Database of Systematic Reviews 2016, Issue 7. Art. No.: CD006663. 10.1002/14651858.CD006663.pub4 PMC6481759 27454104

[8] Moher D, Liberati A, Tetzlaff J, Altman DG. Preferred reporting items for systematic reviews and meta-analyses: The PRISMA statement. BMJ 2009;339:b2535 10.1136/bmj.b2535 19622551 PMC2714657

[9] Stroup DF, Berlin JA, Morton SC, Olkin I, Williamson GD, Rennie D, et al Meta-analysis of observational studies in epidemiology: a proposal for reporting. Meta-analysis Of Observational Studies in Epidemiology (MOOSE) group. JAMA 2000;283:2008–12. 10789670 10.1001/jama.283.15.2008

[10] El Dib R, Nascimento P Junior, Kapoor A. An alternative approach to deal with the absence of clinical trials: a proportional meta-analysis of case series studies. Acta Cir Bras. 2013;28:870–876. 24316861 10.1590/s0102-86502013001200010

[11] El Dib R, Touma NJ, Kapoor A. Cryoablation versus Radiofrequency Ablation for the Treatment of Renal Cell Carcinoma: a meta-analysis of case series studies. BJU Int. 2012a; 110:510–6. 22304329 10.1111/j.1464-410X.2011.10885.x

[12] Lin HY, Liu HC, Huang YH, Liao HC, Hsu TR, Shen CI, et al Effects of enzyme replacement therapy for cardiac-type Fabry patients with a Chinese hotspot late-onset Fabry mutation (IVS4+919G>A). BMJ Open. 2013;3:7. 10.1136/bmjopen-2013-003146 PMC3717460 23864212

[13] Lobo T, Morgan J, Bjorksten A, Nicholls K, Grigg L, Centra E, Becker G. Cardiovascular testing in Fabry disease: exercise capacity reduction, chronotropic incompetence and improved anaerobic threshold after enzyme replacement. Intern Med J. 2008;38:407–14. 10.1111/j.1445-5994.2008.01669.x 18613897

[14] Prabakaran T, Birn H, Bibby BM, Regeniter A, Sørensen SS, Feldt-Rasmussen U, Nielsen R, et al Long-term enzyme replacement therapy is associated with reduced proteinuria and preserved proximal tubular function in women with Fabry disease. Nephrol Dial Transplant. 2014;29:619–25. 10.1093/ndt/gft452 24215016

[15] Rombach SM, Smid BE, Bouwman MG, Linthorst GE, Dijkgraaf MG, Hollak CE. Long term enzyme replacement therapy for Fabry disease: effectiveness on kidney, heart and brain. Orphanet J Rare Dis. 2013;8:47 10.1186/1750-1172-8-47 23531228 PMC3626869

[16] Sirrs SM, Bichet DG, Casey R, Clarke JT, Lemoine K, Doucette S, et al Outcomes of patients treated through the Canadian Fabry disease initiative. Mol Genet Metab. 2014;111:499–506. 10.1016/j.ymgme.2014.01.014 24534763

[17] Anderson LJ, Wyatt KM, Henley W, Nikolaou V, Waldek S, Hughes DA, et al Long-term effectiveness of enzyme replacement therapy in Fabry disease: results from the NCS-LSD cohort study. J Inherit Metab Dis. 2014;37:969–78. 10.1007/s10545-014-9717-4 24831586

[18] Kantola I, Hietaharju A, Taurio J, Kananen K, Kantola T, Viikari J. Quality of life did not worsen for 7 years in enzyme-replacement therapy recipients with fabry disease. Clinical Therapeutics. Conference: 11th European Round Table on Fabry Disease Istanbul Turkey. Conference Start: 20101015 Conference End: 20101016. Conference Publication: (var.pagings). 34 (4 SUPPL. 1) (pp e21-e22), 2012.

[19] Talbot AS, Lewis NT, Nicholls KM. Cardiovascular outcomes in Fabry disease are linked to severity of chronic kidney disease. Heart. 2015;101:287–93. 10.1136/heartjnl-2014-306278 25381325

[20] Rombach SM, Smid BE, Linthorst GE, Dijkgraaf MGW, Hollak CEM. Natural course of Fabry disease and the effectiveness of enzyme replacement therapy: a systematic review and meta- analysis. Journal of Inherited Metabolic Disease 2014; 37:341–52. 10.1007/s10545-014-9677-8 24492980

[21] DerSimonian R, Laird N. Meta-analysis in clinical trials. Control Clin Trials 1986; 7:177–188. 3802833 10.1016/0197-2456(86)90046-2

[22] Feriozzi S, Torras J, Cybulla M, Nicholls K, Sunder-Plassmann G, West M, et al The effectiveness of long-term agalsidase alfa therapy in the treatment of Fabry nephropathy. Clin J Am Soc Nephrol. 2012;7(1):60–9. 10.2215/CJN.03130411 22246281 PMC3265340

[23] Germain DP, Weidemann F, Abiose A, Patel MR, Cizmarik M, Cole JA, et al Analysis of left ventricular mass in untreated men and in men treated with agalsidase-beta: data from the Fabry Registry. Genetics in medicine: official journal of the American College of Medical Genetics. 2013;15(12):958–65. 23703683 10.1038/gim.2013.53

[24] Hughes DA, Barba Romero MA, Hollak CE, Giugliani R, Deegan PB. Response of women with Fabry disease to enzyme replacement therapy: comparison with men, using data from FOS—the Fabry Outcome Survey. Molecular genetics and metabolism. 2011;103(3):207–14. 10.1016/j.ymgme.2011.03.022 21543245

[25] Kampmann C, Perrin A, Beck M. Effectiveness of agalsidase alfa enzyme replacement in Fabry disease: cardiac outcomes after 10 years' treatment. Orphanet journal of rare diseases. 2015;10:125 10.1186/s13023-015-0338-2 26416388 PMC4587871

[26] Kampmann C, Linhart A, Baehner F, Palecek T, Wiethoff CM, Miebach E, et al Onset and progression of the Anderson-Fabry disease related cardiomyopathy. International journal of cardiology. 2008;130(3):367–73. 10.1016/j.ijcard.2008.03.007 18572264

[27] Linthorst GE, Hollak CE, Donker-Koopman WE, Strijland A, Aerts JM. Enzyme therapy for Fabry disease: neutralizing antibodies toward agalsidase alpha and beta. Kidney Int. 2004;66(4):1589–95. 10.1111/j.1523-1755.2004.00924.x 15458455

[28] Schiffmann R, Pastores GM, Lien YH, Castaneda V, Chang P, Martin R, et al Agalsidase alfa in pediatric patients with Fabry disease: a 6.5-year open-label follow-up study. Orphanet J Rare Dis. 2014;9:169 10.1186/s13023-014-0169-6 25425121 PMC4260255

[29] Schiffmann R, Warnock DG, Banikazemi M, Bultas J, Linthorst GE, Packman S, et al Fabry disease: progression of nephropathy, and prevalence of cardiac and cerebrovascular events before enzyme replacement therapy. Nephrol Dial Transplant. 2009;24(7):2102–11. 10.1093/ndt/gfp031 19218538 PMC2698092

[30] Schiffmann R, Askari H, Timmons M, Robinson C, Benko W, Brady RO, et al Weekly enzyme replacement therapy may slow decline of renal function in patients with Fabry disease who are on long-term biweekly dosing. J Am Soc Nephrol. 2007;18(5):1576–83. 10.1681/ASN.2006111263 17409308 PMC1978101

[31] Sirrs SM, Bichet DG, Casey R, Clarke JT, Lemoine K, Doucette S, et al Outcomes of patients treated through the Canadian Fabry disease initiative. Mol Genet Metab 2014;111(4):499–506. 10.1016/j.ymgme.2014.01.014 24534763

[32] Vedder AC, Breunig F, Donker-Koopman WE, Mills K, Young E, Winchester B, et al Treatment of Fabry disease with different dosing regimens of agalsidase: effects on antibody formation and GL-3. Mol Genet Metab. 2008;94(3):319–25. 10.1016/j.ymgme.2008.03.003 18424138

[33] Vedder AC, Linthorst GE, van Breemen MJ, Groener JE, Bemelman FJ, Strijland A, et al The Dutch Fabry cohort: diversity of clinical manifestations and Gb3 levels. J Inherit Metab Dis. 2007;30(1):68–78. 10.1007/s10545-006-0484-8 17206462

[34] West M, Bichet D, Casey R, Clarke J, Sirrs S, LeMoine K. Benefit of enzyme replacement therapy in Fabry disease: Comparison of outcomes in the Canadian fabry disease initiative study.Molecular Genetics and Metabolism. Conference: 9th Annual Research Meeting of the Lysosomal Disease Network, WORLD Symposium, 2013 Orlando, FL United States. Conference Start: 20130212 Conference End: 20130215. Conference Publication: (var.pagings). 108 (2) (pp S97), 2013.

[35] Arash-Kaps L, Wachter M, Karabul N, Beck M, Kampmann C, Mengel E, et al. 10 years therapy with agalsidase A: Prevention of renal failure in male Fabry patients. Journal of Inherited Metabolic Disease. Conference: Annual Symposium of the Society for the Study of Inborn Errors of Metabolism, SSIEM 2014 Innsbruck Austria. Conference Start: 20140902 Conference End: 20140905. Conference Publication: (var.pagings). 37 (1 SUPPL. 1) (pp S145), 2014.

[36] Baehner F, Kampmann C, Whybra C, Miebach E, Wiethoff CM, Beck M. Enzyme replacement therapy in heterozygous females with Fabry disease: results of a phase IIIB study. J Inherit Metab Dis. 2003;26:617–27. 14707510 10.1023/b:boli.0000005658.14563.77

[37] Barba-Romero MÁ, Rivera-Gallego A, Pintos-Morell G; Spanish FOS-Study Group. Fabry disease in Spain: description of Spanish patients and a comparison with other European countries using data from the Fabry Outcome Survey (FOS). Int J Clin Pract. 2011;65(8):903–10. 10.1111/j.1742-1241.2011.02695.x 21679285

[38] Beer M, Weidemann F, Breunig F, Knoll A, Koeppe S, Machann W, et al Impact of enzyme replacement therapy on cardiac morphology and function and late enhancement in Fabry's cardiomyopathy. Am J Cardiol. 2006;97(10):1515–8. 10.1016/j.amjcard.2005.11.087 16679096

[39] Borgwardt L, Feldt-Rasmussen U, Rasmussen AK, Ballegaard M, Meldgaard Lund A. Fabry disease in children: agalsidase-beta enzyme replacement therapy. Clin Genet. 2013;83(5):432–8. 10.1111/j.1399-0004.2012.01947.x 22880956

[40] Collin C, Briet M, Tran TC, Beaussier H, Benistan K, Bensalah M, et al Long-term changes in arterial structure and function and left ventricular geometry after enzyme replacement therapy in patients affected with Fabry disease. Eur J Prev Cardiol. 2012;19(1):43–54. 10.1177/1741826710391118 21450622

[41] Dehout FDR, Granseigne ST, Guillauillaume B, Van Maldergem L. Relief of gastrointestinal symptoms under enzyme replacement therapy in patients with Fabry disease. Inherit Metab Dis. 2004;27:499–505. 10.1023/B:BOLI.0000037342.59612.69 15303007

[42] Donati D, Novario R, Gastaldi L. Natural history and treatment of uremia secondary to Fabry's disease: an European experience. Nephron. 1987;46(4):353–9. 3116442 10.1159/000184389

[43] McKechine Douglas GJ, Mac Lochlainn Dylan J, Metha Atul B., Hughes Derralynn A. Long term clinical outcomes in patients with Fabry disease receiving enzyme replacement therapy. Molecular Genetics and Metabolism 114(2015) S11–S130. 10.1016/j.ymgme.2017.10.001 29055531

[44] Elliott PM, Kindler H, Shah JS, Sachdev B, Rimoldi OE, Thaman R, et al Coronary microvascular dysfunction in male patients with Anderson-Fabry disease and the effect of treatment with alpha galactosidase A. Heart. 2006;92(3):357–60. 10.1136/hrt.2004.054015 16085718 PMC1860797

[45] Gallego RA, Rodríguez ML, Hernández FJB, Romero MAB, Mateos AGL, Morelle GP. Enfermedad de Fabry en España: primer análisis de la respuesta al tratamiento de sustitución enzimática. Medicina Clínica. 2006;127(13):481–4. 17043001 10.1157/13093265

[46] Guffon N, Fouilhoux A. NGaA. Clinical benefit in Fabry patients given enzyme replacement therapy—A case series. J Inherit MetabDis. 2004;27:221–7. 10.1023/B:BOLI.0000028726.11177.8b 15159653

[47] Guffon N. Clinical presentation in female patients with Fabry disease. J Med Genet 2003; 40:e38 10.1136/jmg.40.4.e38 12676911 PMC1735431

[48] Hoffmann B, Schwarz M, Mehta A, Keshav S. Fabry outcome survey European I. Gastrointestinal symptoms in 342 patients with Fabry disease: prevalence and response to enzyme replacement therapy. Clin Gastroenterol Hepatol. 2007;5:1447–53. 10.1016/j.cgh.2007.08.012 17919989

[49] Hopkin R, Mauer M, Lemay R, Strotmann J, Sims K. Fabry registry data indicate that early initiation of agalsidase beta treatment is associated with fewer clinical events. Molecular Genetics and Metabolism. Conference: 8th Annual Research Meeting of the Lysosomal Disease Network, WORLD Symposium 2012 San Diego, CA United States. Conference Start: 20120207 Conference End: 20120210. Conference Publication: (var.pagings). 105 (2) (pp S36), 2012.

[50] Hopkin R, Mauer M, Lemay R, Ortiz J, Sims K, Waldek S. Early initiation of agalsidase beta treatment is associated with fewer clinical events in women with Fabry disease: Data from the Fabry Registry. Molecular genetics and metabolism. 2013;108(2):S49.

[51] Imbriaco M, Pisani A, Spinelli L, Cuocolo A, Messalli G, Capuano E, et al Effects of enzyme-replacement therapy in patients with Anderson-Fabry disease: a prospective long-term cardiac magnetic resonance imaging study. Heart. 2009;95(13):1103–7. 10.1136/hrt.2008.162800 19372091

[52] Kalliokoski RJ, Kantola I, Kalliokoski KK, Engblom E, Sundell J, Hannukainen JC, et al The effect of 12-month enzyme replacement therapy on myocardial perfusion in patients with Fabry disease. J Inherit Metab Dis. 2006;29(1):112–8. 10.1007/s10545-006-0221-3 16601877

[53] Koskenvuo JW, Hartiala JJ, Nuutila P, Kalliokoski R, Viikari JS, Engblom E, Penttinen M, Knuuti J, Mononen I, Kantola IM.Twenty-four-month alpha-galactosidase A replacement therapy in Fabry disease has only minimal effects on symptoms and cardiovascular parameters.J Inherit Metab Dis. 2008;31(3):432–41. 10.1007/s10545-008-0848-3 18509742

[54] Linhart A, Kampmann C, Zamorano JL, Sunder-Plassmann G, Beck M, Mehta A, Elliott PM; European FOS Investigators. Cardiac manifestations of Anderson Fabry disease: results from the international Fabry outcome survey.Eur Heart J. 2007;28(10):1228–35. 10.1093/eurheartj/ehm153 17483538

[55] Lubanda JC, Anijalg E, Bzdúch V, Thurberg BL, Bénichou B, Tylki-Szymanska A. Evaluation of a low dose, after a standard therapeutic dose, of agalsidase beta during enzyme replacement therapy in patients with Fabry disease. Genet Med. 2009;11(4):256–64. 10.1097/GIM.0b013e3181981d82 19265719

[56] MacDermot KD, Holmes A, Miners AH. Anderson-Fabry disease: clinical manifestations and impact of disease in a cohort of 60 obligate carrier females. J Med Genet. 2001;38(11):769–75. 10.1136/jmg.38.11.769 11732485 PMC1734754

[57] Motwani M, Banypersad S, Woolfson P, Waldek S. Enzyme replacement therapy improves cardiac features and severity of Fabry disease.Mol Genet Metab. 2012;107(1–2):197–202. 10.1016/j.ymgme.2012.05.011 22704481

[58] Parini R, Rigoldi M, Santus F, Furlan F, De Lorenzo P, Valsecchi G, et al Enzyme replacement therapy with agalsidase alfa in a cohort of Italian patients with Anderson-Fabry disease: testing the effects with the Mainz Severity Score Index. Clin Genet. 2008;74(3):260–6. 10.1111/j.1399-0004.2008.01012.x 18445046

[59] Pisani A, Spinelli L, Sabbatini M, Andreucci MV, Procaccini D, Abbaterusso C, et al Enzyme replacement therapy in Fabry disease patients undergoing dialysis: effects on quality of life and organ involvement. Am J Kidney Dis. 2005;46(1):120–7. 15983965 10.1053/j.ajkd.2005.03.016

[60] Rombach SM, Smid BE, Bouwman MG, Linthorst GE, Dijkgraaf MG, Hollak CE. Long term enzyme replacement therapy for Fabry disease: effectiveness on kidney, heart and brain. Orphanet J Rare Dis. 2013; 25;8:47 10.1186/1750-1172-8-47 23531228 PMC3626869

[61] Spinelli L, Pisani A, Sabbatini M, Petretta M, Andreucci MV, Procaccini D, et al Enzyme replacement therapy with agalsidase beta improves cardiac involvement in Fabry's disease. Clin Genet 2004;66(2):158–65. 10.1111/j.1399-0004.2004.00284.x 15253767

[62] Weidemann F, Niemann M, Störk S, Breunig F, Beer M, Sommer C, et al Long-term outcome of enzyme-replacement therapy in advanced Fabry disease: evidence for disease progression towards serious complications. J Intern Med. 2013;274(4):331–41. 10.1111/joim.12077 23586858 PMC4282332

[63] Whybra C, Miebach E, Mengel E, Gal A, Baron K, Beck M, et al A 4-year study of the efficacy and tolerability of enzyme replacement therapy with agalsidase alfa in 36 women with Fabry disease. Genet Med. 2009;11(6):441–9. 10.1097/GIM.0b013e3181a23bec 19346951

[64] Cybulla M, West M, Nicholls K, Torras J, Sunder-Plassmann G, Feriozzi S. Effectiveness and safety of enzyme replacement therapy (ERT) in acohort of kidney transplant recipients with fabry disease. Nephrology Dialysis Transplantation. Conference: 50th ERA-EDTA Congress Istanbul Turkey. Conference Start: 20130518 Conference End: 20130521. Conference Publication: (var.pagings). 28 (pp i287), 2013.

[65] Eng CM, Fletcher J, Wilcox WR, Waldek S, Scott CR, Sillence DO, et al Fabry disease: baseline medical characteristics of a cohort of 1765 males and females in the Fabry Registry. J Inherit Metab Dis. 2007;30(2):184–92. 10.1007/s10545-007-0521-2 17347915

[66] Hilz MJ, Marthol H, Schwab S, Kolodny EH, Brys M, Stemper B. Enzyme replacement therapy improves cardiovascular responses to orthostatic challenge in Fabry patients. J Hypertens. 2010;28(7):1438–48. 10.1097/HJH.0b013e328336a077 20125036

[67] Mehta A, Beck M, Elliott P, Giugliani R, Linhart A, Sunder-Plassmann G, Schiffmann R, Barbey F, Ries M, Clarke JT; Fabry Outcome Survey investigators. Enzyme replacement therapy with agalsidase alfa in patients with Fabry's disease: an analysis of registry data. Lancet. 2009 12;374(9706):1986–96. 10.1016/S0140-6736(09)61493-8 19959221

[68] Patel MR, Cecchi F, Cizmarik M, Kantola I, Linhart A, Nicholls K, et al Cardiovascular events in patients with fabry disease natural history data from the fabry registry.J Am Coll Cardiol. 2011;57(9):1093–9. 10.1016/j.jacc.2010.11.018 21349401

[69] Ramaswami U, Parini R, Pintos-Morell G, Kalkum G, Kampmann C, Beck M; FOS Investigators. Fabry disease in children and response to enzyme replacement therapy: results from the Fabry Outcome Survey. Clin Genet. 2012;81(5):485–90. 10.1111/j.1399-0004.2011.01671.x 21457233

[70] Ries M, Clarke JT, Whybra C, Timmons M, Robinson C, Schlaggar BL, et al Enzyme-replacement therapy with agalsidase alfa in children with Fabry disease.Pediatrics. 2006;118(3):924–32. 10.1542/peds.2005-2895 16950982

[71] Schwarting A, Dehout F, Feriozzi S, Beck M, Mehta A, Sunder-Plassmann G; et al Enzyme replacement therapy and renal function in 201 patients with Fabry disease. Clin Nephrol. 2006;66(2):77–84. 16939062

[72] Sims K, Politei J, Banikazemi M, Lee P. Stroke in Fabry disease frequently occurs before diagnosis and in the absence of other clinical events: natural history data from the Fabry Registry. Stroke. 2009;40(3):788–94. 10.1161/STROKEAHA.108.526293 19150871

[73] Warnock DG, Ortiz A, Mauer M, Linthorst GE, Oliveira JP, Serra AL, et al Renal outcomes of agalsidase beta treatment for Fabry disease: role of proteinuria and timing of treatment initiation. Nephrol Dial Transplant. 2012;27(3):1042–9. 10.1093/ndt/gfr420 21804088 PMC3289896

[74] Watt T, Burlina AP, Cazzorla C, Schönfeld D, Banikazemi M, Hopkin RJ, et al Agalsidase beta treatment is associated with improved quality of life in patients with Fabry disease: findings from the Fabry Registry. Genet Med. 2010;12(11):703–12. 10.1097/GIM.0b013e3181f13a4a 20885332

[75] Wraith JE, Tylki-Szymanska A, Guffon N, Lien YH, Tsimaratos M, Vellodi A, et al Safety and efficacy of enzyme replacement therapy with agalsidase beta: an international, open-label study in pediatric patients with Fabry disease. J Pediatr. 2008;152(4):563–70, 570.e1 10.1016/j.jpeds.2007.09.007 18346516

[76] Wu JC, Ho CY, Skali H, Abichandani R, Wilcox WR, Banikazemi M, et al Cardiovascular manifestations of Fabry disease: relationships between left ventricular hypertrophy, disease severity, and alpha-galactosidase A activity. Eur Heart J. 2010;31(9):1088–97. 10.1093/eurheartj/ehp588 20061327 PMC2912636

[77] Branton MH, Schiffmann R, Sabnis SG, Murray GJ, Quirk JM, Altarescu G, et al Natural history of Fabry renal disease: influence of alpha-galactosidase A activity and genetic mutations on clinical course. Medicine. 2002;81(2):122–38. 11889412 10.1097/00005792-200203000-00003

[78] Crutchfield KE, Patronas NJ, Dambrosia JM, Frei KP, Banerjee TK, Barton NW, Schiffmann R. Quantitative analysis of cerebral vasculopathy in patients with Fabry disease. Neurology. 1998;50(6):1746–9. 9633721 10.1212/wnl.50.6.1746

[79] Hebert A, Lacbawan L, Taber T, Goker-Alpan O. Evaluation of long-term enzyme replacement therapy for children with Fabry disease. Molecular Genetics and Metabolism. Conference: 9th Annual Research Meeting of the Lysosomal Disease Network, WORLD Symposium, 2013 Orlando, FL United States. Conference Start: 20130212 Conference End: 20130215. Conference Publication: (var.pagings). 108 (2) (pp S47), 2013.

[80] Ortiz A, Abiose A, Bichet DG, Cabrera G, Charrow J, Germain DP, et al Time to treatment benefit for adult patients with Fabry disease receiving agalsidase β: data from the Fabry Registry. J Med Genet. 2016 3 18. pii: jmedgenet-2015-103486. 10.1136/jmedgenet-2015-103486 PMC4941144 26993266

[81] Ortiz A, Cianciaruso B, Cizmarik M, Germain DP, Mignani R, Oliveira JP, et al End-stage renal disease in patients with Fabry disease: natural history data from the Fabry Registry. Nephrol Dial Transplant. 2010 3;25(3):769–75. 10.1093/ndt/gfp554 19846394

[82] Pano A, Goker-Alpan O, Longo N, McDonald M, Shankar S, Shen Y, Chang P, Schiffmann R. Safety and effect of open-label agalsidase alfa in treatment-naive children with Fabry disease. Molecular Genetics and Metabolism. Conference: 10th Annual Research Meeting of the Lysosomal Disease Network, WORLD Symposium, 2014 San Diego, CA United States. Conference Start: 20140210 Conference End: 20140213. Conference Publication: (var.pagings). 111 (2) (pp S84), 2014.

[83] Pastores GM, Boyd E, Crandall K, Whelan A, Piersall L, Barnett N. Safety and pharmacokinetics of agalsidase alfa in patients with Fabry disease and end-stage renal disease. Nephrol Dial Transplant. 2007;22(7):1920–5. 10.1093/ndt/gfm096 17395657

[84] Choi JH, Cho YM, Suh KS, Yoon HR, Kim GH, Kim SS, et al Short-term efficacy of enzyme replacement therapy in Korean patients with Fabry disease. J Korean Med Sci. 2008;23(2):243–50. 10.3346/jkms.2008.23.2.243 18437007 PMC2526436

[85] Eto Y, Ohashi T, Utsunomiya Y, Fujiwara M, Mizuno A, Inui K, et al Enzyme replacement therapy in Japanese Fabry disease patients: the results of a phase 2 bridging study. J Inherit Metab Dis. 2005;28:575–83. 10.1007/s10545-005-0575-y 15902561

[86] Fujiwara M, Ohashi T, Kobayashi M, Hiroyuki I, Yoshikatsu E. The cardiac effects of enzyme replacement therapy for fabry disease: Comparison of clinical course between female and male patients. Tokyo Jikeikai Medical Journal. 122 (6) (pp 295–304), 2007.

[87] Goto H, Tsuboi K, Yamamoto H. Cardiac manifestations and enzyme replacement therapy of Fabry disease. Journal of Inherited Metabolic Disease. Conference: 12th International Congress of Inborn Errors of Metabolism, ICIEM 2013 Barcelona Spain. Conference Start: 20130903 Conference End: 20130906. Conference Publication: (var.pagings). 36 (2 SUPPL. 1) (pp S261), 2013.

[88] Kim JH, Cho JH, Lee BH, Choi JH, Yoo HW. Long-term efficacy of enzyme replacement therapy (ERT) for Fabry disease: Experience of single institution. Journal of Inherited Metabolic Disease. Conference: Annual Symposium of the Society for the Study of Inborn Errors of Metabolism, SSIEM 2015 Lyon France. Conference Start: 20150901 Conference End: 20150904. Conference Publication: (var.pagings). 38 (1 SUPPL. 1) (pp S265-S266), 2015.

[89] Kobayashi M, Ohashi T, Sakuma M, Ida H, Eto Y. Clinical manifestations and natural history of Japanese heterozygous females with Fabry disease. J Inherit Metab Dis. 2008;31 Suppl 3:483–7. 18202903 10.1007/s10545-007-0740-6

[90] Kobayashi M, Ida H, Ohashi T, Eto Y. Safety of enzyme replacement therapy among 20 japanese patients with classical type of fabry disease. J Inherit Metab Dis 2005; 28(suppl 1).

[91] Tsuboi K, Yamamoto H, Goto H. Efficacy of enzyme replacement therapy with agalsidase alfa for 32 naive Fabry disease patients. Journal of Inherited Metabolic Disease. Conference: Annual Symposium of the Society for the Study of Inborn Errors of Metabolism, SSIEM 2015 Lyon France. Conference Start: 20150901 Conference End: 20150904. Conference Publication: (var.pagings). 38 (1 SUPPL. 1) (pp S291-S292), 2015a.

[92] Tsuboi K, Yamamoto H, Goto H, Ota A. Efficacy of enzyme replacement therapy with agalsidase beta for 17 naive Fabry disease patients. Journal of Inherited Metabolic Disease. Conference: Annual Symposium of the Society for the Study of Inborn Errors of Metabolism, SSIEM 2015 Lyon France. Conference Start: 20150901 Conference End: 20150904. Conference Publication: (var.pagings). 38 (1 SUPPL. 1) (pp S292), 2015b.

[93] Domínguez RO, Amartino H, Chamoles NA, Grupo de Estudio de la Enfermedad de Fabry. Dolor neuropático en la enfermedad de Fabry: remisión heterogénea en tres años de tratamiento de reemplazo enzimático. Rev Neurol 2006;43:201–206. 16883508

[94] Jardim LB, Gomes I, Netto CB, Nora DB, Matte US, Pereira F, et al Improvement of sympathetic skin responses under enzyme replacement therapy in Fabry disease. J Inherit Metab Dis. 2006;29(5):653–9. 10.1007/s10545-006-0339-3 16972173

[95] Kisinovsky I C G, Coronel C, Reisin R. Home infusion program for Fabry disease: experience with agalsidase alfa in Argentina. MEDICINA 2013;73(1):31–4. 23335703

[96] Martins AM, Aranda P, Biagini G, Derossi L, Rosa M, Munoz-Rojas MV. Betagalsidase safety: Brazilian experience with patients under 16 years of age. J.ymgme. 2014 12 170.

[97] Martins AM, Kyosen SO, Garrote J, Marques FM, Guilhem JG, Macedo E, et al Demographic characterization of Brazilian patients enrolled in the Fabry Registry. Genet Mol Res. 2013;12(1):136–42. 10.4238/2013.January.24.5 23408399

[98] Thofehrn S, Netto C, Cecchin C, Burin M, Matte U, Brustolin S, et al Kidney Function and 24-Hour Proteinuria in Patients with Fabry Disease during 36 Months of Agalsidase Alfa Enzyme Replacement Therapy: A Brazilian Experience. Ren Fail 2009;31(9):773–8. 10.3109/08860220903150296 19925283

[99] Johnson FK, Valenzano K, Castelli J. Comparison of integrated white blood cell alpha-galactosidase a activity exposure between every-other-day orally administered migalastat and biweekly infusions of agalsidase beta or agalsidase alfa Mol Genet Metab 2016;117:S63.

[100] Tøndel C, Bostad L, Larsen KK, Hirth A, Vikse BE, Houge G, et al Agalsidase benefits renal histology in young patients with Fabry disease. J Am Soc Nephrol. 2013;24:137–48. 10.1681/ASN.2012030316 23274955 PMC3537211

[101] West M, Bichetb DG, Caseyc R, Clarked JTR, Iwanochkoe M, Khan A, et al. Canadian Fabry Disease Initiative Study (CFDI): 8 year outcomes of a randomized controlled trial of enzyme replacement therapy (ERT). http://garrodsymposium.com/garrod2016/posters/#p104. Accessed on July 18, 2016.

[102] Lin DY, Sullivan PF. Meta-analysis of genome-wide association studies with overlapping subjects. Am J Hum Genet. 2009;85:862–72. 10.1016/j.ajhg.2009.11.001 20004761 PMC2790578

[103] Banikazemi M, Bultas J, Waldek S, Wilcox WR, Whitley CB, McDonald M, et al Agalsidase-beta therapy for advanced Fabry disease: a randomized trial. Ann Intern Med. 2007 1 16;146(2):77–86. 17179052 10.7326/0003-4819-146-2-200701160-00148

[104] El Dib RP, Atallah AN, Andriolo RB. Mapping the Cochrane evidence for decision making in health care. J Eval Clin Pract 2007;13:689–92. 10.1111/j.1365-2753.2007.00886.x 17683315

[105] Villas Boas PJ, Spagnuolo RS, Kamegasawa A, Braz LG, Polachini do Valle A, Jorge EC, et al Systematic reviews showed insufficient evidence for clinical practice in 2004: what about in 2011? The next appeal for the evidence-based medicine age. J Eval Clin Pract 2013;19:633–7. 10.1111/j.1365-2753.2012.01877.x 22747638

[106] Cook DJ, Mulrow CD, Haynes RB. Systematic reviews: synthesis of best evidence for clinical decisions. Ann Intern Med 1997;126:376–80. 9054282 10.7326/0003-4819-126-5-199703010-00006

[107] Alderson P, Chalmers I. Survey of claims of no effect in abstracts of Cochrane reviews. BMJ 2003;326:475 10.1136/bmj.326.7387.475 12609942 PMC150179

[108] Shemesh E, Deroma L, Bembi B, Deegan P, Hollak C, Weinreb NJ, et al Enzyme replacement and substrate reduction therapy for Gaucher disease. Cochrane Database of Systematic Reviews 2015, Issue 3. Art. No.: CD010324. 10.1002/14651858.CD010324.pub2 PMC8923052 25812601

