# Supplementary material for: Enzyme replacement therapy for Anderson-Fabry disease: A complementary overview of a Cochrane publication through a linear regression and a pooled analysis of proportions from cohort studies
Source: PLoS One. 2017 Mar 15;12(3):e0173358. doi: 10.1371/journal.pone.0173358 (PMC5351840; doi:10.1371/journal.pone.0173358)
Supplement: S2 Table — (DOCX) [file pone.0173358.s002.docx]

**S2 Table.** Search strategy.

| (Fabry Disease OR Angiokeratoma Corporis Diffusum OR Anderson-Fabry Disease OR Anderson Fabry Disease OR Fabry's Disease OR Anderson Fabry's Disease OR Fabrys Disease OR Anderson Fabrys Disease OR AFD OR alpha-Galactosidase A Deficiency Disease OR alpha Galactosidase A Deficiency Disease OR Angiokeratoma Corporis Diffusum OR Angiokeratoma Diffuse OR Ceramide Trihexosidase Deficiency OR GLA Deficiency OR Hereditary Dystopic Lipidosis OR alpha-Galactosidase A Deficiency OR alpha Galactosidase A Deficiency OR Diffuse Angiokeratoma OR Classic Fabry disease OR Non classic Fabry disease OR Non-classic Fabry disease OR Classic Anderson Fabry disease OR Non classic Anderson Fabry disease OR Non-classic Anderson Fabry disease OR Late onset Fabry disease OR Late-onset Fabry disease OR Late onset Anderson Fabry disease OR Late-onset Anderson Fabry disease) AND (agalsidase beta OR Fabrazyme OR Genzyme brand of AGAL OR Agalsidase alfa OR Agalsidase alpha OR Replagal OR Shire HGT brand of AGAL OR alpha Galactosidase OR alpha-Galactosidases OR alpha Galactosidases OR Melibiase OR alpha-Galactosidase A OR alpha Galactosidase A OR Beano OR enzyme replacement therapy OR enzyme replacement therapies OR ERT OR natural history OR untreated OR untreated patients OR no ERT OR healthy control OR healthy volunteers OR placebo OR no intervention) |
| --- |
