# Supplementary material for: Enzyme replacement therapy for Anderson-Fabry disease: A complementary overview of a Cochrane publication through a linear regression and a pooled analysis of proportions from cohort studies
Source: PLoS One. 2017 Mar 15;12(3):e0173358. doi: 10.1371/journal.pone.0173358 (PMC5351840; doi:10.1371/journal.pone.0173358)
Supplement: S3 Table — (DOCX) [file pone.0173358.s003.docx]

**S3 Table.** Information about contact with authors of the included studies that replied to request on whether there was an overlap of patients in multiple publications, and reasons to include or not these publications.

| Author, year | References of multiple publications | Responses from the authors of the included studies about whether there is an overlap of patients in their publications | Reasons on whether to include or not these publications |
| --- | --- | --- | --- |
| Kampmann 2015 [25] and 2008 [26] | - Kampmann C, Perrin A, Beck M. Effectiveness of agalsidase alfa enzyme replacement in Fabry disease: cardiac outcomes after 10 years' treatment. Orphanet journal of rare diseases. 2015;10:125.  - Kampmann C, Linhart A, Baehner F, Palecek T, Wiethoff CM, Miebach E, et al. Onset and progression of the Anderson-Fabry disease related cardiomyopathy. International journal of cardiology. 2008;130(3):367-73.  - Mehta A, Ricci R, Widmer U, Dehout F, Garcia de Lorenzo A, Kampmann C, et al. Fabry disease defined: baseline clinical manifestations of 366 patients in the Fabry Outcome Survey. Eur J Clin Invest. 2004;34(3):236-42. | No overlap. | To include both publications as Kampmann 2015 [34] study evaluated the effects of agalsidase alfa, and Kampmann 2008 [35] study evaluated natural history of FD patients. Mehta 2004 is the same database from Kampmann studies. |
| Schiffmann 2014 [28], 2009 [29], and 2007 [30] | - Schiffmann R, Swift C, Wang X, Blankenship D, Ries M. A prospective 10-year study of individualized, intensified enzyme replacement therapy in advanced Fabry disease. J Inherit Metab Dis. 2015; 38(6):1129-36.  - Schiffmann R, Pastores GM, Lien YH, Castaneda V, Chang P, Martin R, et al. Agalsidase alfa in pediatric patients with Fabry disease: a 6.5-year open-label follow-up study.Orphanet J Rare Dis. 2014; 9:169.  - Schiffmann R, Ries M, Blankenship D, Nicholls K, Mehta A, Clarke JT, et al. Changes in plasma and urine globotriaosylceramide levels do not predict Fabry disease progression over 1 year of agalsidase alfa. Genet Med. 2013; 15(12):983-9.  - Schiffmann R, Martin RA, Reimschisel T, Johnson K, Castaneda V, Lien YH, et al. Four-year prospective clinical trial of agalsidase alfa in children with Fabry disease. J Pediatr. 2010; 156(5):832-7, 837.e1.  - Schiffmann R, Warnock DG, Banikazemi M, Bultas J, Linthorst GE, Packman S, et al. Fabry disease: progression of nephropathy, and prevalence of cardiac and cerebrovascular events before enzyme replacement therapy. Nephrol Dial Transplant. 2009 Jul;24(7):2102-11.  - Schiffmann R, Askari H, Timmons M, Robinson C, Benko W, Brady RO, et al. Weekly enzyme replacement therapy may slow decline of renal function in patients with Fabry disease who are on long-term biweekly dosing. J Am Soc Nephrol. 2007; 18(5):1576-83.  - Schiffmann R, Ries M, Timmons M, Flaherty JT, Brady RO. Long-term therapy with agalsidase alfa for Fabry disease: safety and effects on renal function in a home infusion setting. Nephrol Dial Transplant. 2006; 21(2):345-54.  - Schiffmann R, Floeter MK, Dambrosia JM, Gupta S, Moore DF, Sharabi Y, Khurana RK, et al. Enzyme replacement therapy improves peripheral nerve and sweat function in Fabry disease. Muscle Nerve. 2003; 28(6):703-10.  - Schiffmann R. Natural history of Fabry disease in males: preliminary observations. J Inherit Metab Dis. 2001;24 Suppl 2:15-7 | Patients may have overlapped. | To include Schiffmann 2014 [59] as the authors evaluated pediatric FD patients, and Schiffmann 2007 [61] as it was analysed adults hemizygous male patients and, Schiffmann 2009 [60].  Schiffmann 2001 was a short report and did not report the sample size of the patients evaluated, therefore we did not use this study to collect data.  Schiffmann 2015 used a non-standard dose for agalsidase alfa (0.4 mg/kg) therefore we did not use this study in the meta-analysis. |
| Sirrs 2014 [31] | - Sirrs S, Clarke JT, Bichet DG, Casey R, Lemoine K, Flowerdew G, et al. Baseline characteristics of patients enrolled in the Canadian Fabry Disease Initiative. Mol Genet Metab. 2010; 99(4):367-73.  - Sirrs SM, Bichet DG, Casey R, Clarke JT, Lemoine K, Doucette S, et al. Outcomes of patients treated through the Canadian Fabry disease initiative. Mol Genet Metab. 2014 Apr;111(4):499-506. | Canadian Fabry disease initiative, patients may have overlapped. | We only considered cohort 1c from the Sirrs 2014 [] study because it presented patients naive to ERT who do not meet ERT criteria or who do not consent to ERT. Cohort 1a was excluded as it presented patients that swichted from different ERT regimens and, we also excluded cohort 1b because this was a randomized controlled trial.  Sirrs 2010 study presented fewer number of patients compared to Sirrs 2014 [86] study. |
| Germain 2013 [23] | - Germain DP, Charrow J, Desnick RJ, Guffon N, Kempf J, Lachmann RH, et al. Ten-year outcome of enzyme replacement therapy with agalsidase beta in patients with Fabry disease. Journal of medical genetics. 2015;52(5):353-8.  - Wilcox W, Feldt-Rasmussen U, Maria Martins A, Ortiz A, Weidemann F, Lemay R, et al. Female Fabry disease: Significant improvement of Fabry disease-related gastrointestinal symptoms in a large cohort of female patients treated with agalsidase beta: Data from the Fabry Registry. Molecular Genetics and Metabolism. Conference: 10th Annual Research Meeting of the Lysosomal Disease Network, WORLD Symposium, 2014 San Diego, CA United States. Conference Start: 20140210 Conference End: 20140213. Conference Publication: 111 (2) (pp S114-S115), 2014.  - Germain DP, Weidemann F, Abiose A, Patel MR, Cizmarik M, Cole JA, et al. Analysis of left ventricular mass in untreated men and in men treated with agalsidase-beta: data from the Fabry Registry. Genetics in medicine: official journal of the American College of Medical Genetics. 2013;15(12):958-65.  - Thurberg BL, Fallon JT, Mitchell R, Aretz T, Gordon RE, O'Callaghan MW. Cardiac microvascular pathology in Fabry disease: evaluation of endomyocardial biopsies before and after enzyme replacement therapy. Circulation. 2009 May 19;119(19):2561-7.  - Germain DP, Waldek S, Banikazemi M, Bushinsky DA, Charrow J, Desnick RJ, et al. Sustained, long-term renal stabilization after 54 months of agalsidase beta therapy in patients with Fabry disease. J Am Soc Nephrol 2007; 18(5):1547-57. | Fabry Registry, patients may have overlapped. | To include Germain 2013 [21] study as the main publication because of the numbers of patients being greater than in the other publications, and because there was also an untreated control group. However, we also considered the publications from Germain 2015 and Wilcox 2014 studies for the data on patient-important outcomes. |
| West 2013 [34] | - West M, Bichet D, Casey R, Clarke J, Sirrs S, LeMoine K. Benefit of enzyme replacement therapy in Fabry disease: Comparison of outcomes in the Canadian fabry disease initiative study. Molecular Genetics and Metabolism. Conference: 9th Annual Research Meeting of the Lysosomal Disease Network, WORLD Symposium, 2013 Orlando, FL United States. Conference Start: 20130212 Conference End: 20130215. Conference Publication: 108 (2) (pp S97), 2013.  - West M, Nicholls K, Mehta A, Clarke JT, Steiner R, Beck M, et al. Agalsidase alfa and kidney dysfunction in Fabry disease. J Am Soc Nephrol. 2009; 20(5):1132-9.  - West M, Bichet D, Casey R, Clarke J, Sirrs S, LeMoine K. Agalsidase alpha and agalsidase beta have similar effects on outcomes in fabry disease: Results from the Canadian fabry disease initiative. Molecular Genetics and Metabolism 102 (2011) S3–S47 (abstract). | Canadian Fabry Disease Initiative, patients may have overlapped. | To include West 2013 [74] study; although it is available as abstract the authors compared agalsidase alfa versus beta, and West 2009 study involved fewer patients than West 2013 [74] study. |
| Feriozzi 2012 [22] | - Feriozzi S, Torras J, Cybulla M, Nicholls K, Sunder-Plassmann G, West M, et al. The effectiveness of long-term agalsidase alfa therapy in the treatment of Fabry nephropathy. Clin J Am Soc Nephrol. 2012;7(1):60-9.  - Feriozzi S, Schwarting A, Sunder-Plassmann G, West M, Cybulla M; International Fabry Outcome Survey Investigators. Agalsidase alfa slows the decline in renal function in patients with Fabry disease. Am J Nephrol. 2009;29(5):353-61. | Patients do not overlap. | To include Feriozzi 2012 [18] study as both publications presented very similar data and objectives. |
| Hughes 2011 [24] | - Hughes D, Ramaswami U, Mckie M, Dimson E, Baker R, Ebrahim H, et al. Fabry disease: Impact of ERT on renal function. Single-center 5-year results. Molecular Genetics and Metabolism. Conference: 11th Annual Research Meeting of the Lysosomal Disease Network, WORLD Symposium, 2015 Orlando, FL United States. Conference Start: 20150209 Conference End: 20150213. Conference Publication: 114 (2) (pp S56-S57), 2015.  - Hughes DA, Barba Romero MA, Hollak CE, Giugliani R, Deegan PB. Response of women with Fabry disease to enzyme replacement therapy: comparison with men, using data from FOS--the Fabry Outcome Survey. Molecular genetics and metabolism. 2011;103(3):207-14. | Fabry Outcome Survey, patients may have overlapped. | To include Hughes 2011 [30] study as this was available in full-text. |
| Vedder 2008 [32] and 2007 [33] | - Vedder AC, Breunig F, Donker-Koopman WE, Mills K, Young E, Winchester B, et al. Treatment of Fabry disease with different dosing regimens of agalsidase: effects on antibody formation and GL-3. Mol Genet Metab. 2008; 94(3):319-25.  - Vedder AC, Linthorst GE, van Breemen MJ, Groener JE, Bemelman FJ, Strijland A, et al. The Dutch Fabry cohort: diversity of clinical manifestations and Gb3 levels. J Inherit Metab Dis. 2007; 30(1):68-78. | No overlap. | To include both publications as Vedder 2008 [69] study evaluated the effects of agalsidase alfa while Vedder 2007 [70] study evaluated only natural history from FD patients. |
| Linthorst 2004 [27] | - Linthorst GE, Vedder AC, Ormel EE, Aerts JM, Hollak CE. Home treatment for Fabry disease: practice guidelines based on 3 years experience in The Netherlands. Nephrol Dial Transplant 2006;21: 355–360.  - Linthorst GE, Hollak CE, Donker-Koopman WE, Strijland A, Aerts JM. Enzyme therapy for Fabry disease: neutralizing antibodies toward agalsidase alpha and beta. Kidney Int. 2004; 66(4):1589-95. | Same Dutch Fabry cohort, patients may have overlapped. | To include Linthorst 2004 [42] study as Linthorst 2006 study was a guideline. |

ERT: enzyme replacement therapy; FD: Fabry disease.
