# Supplementary material for: Enzyme replacement therapy for Anderson-Fabry disease: A complementary overview of a Cochrane publication through a linear regression and a pooled analysis of proportions from cohort studies
Source: PLoS One. 2017 Mar 15;12(3):e0173358. doi: 10.1371/journal.pone.0173358 (PMC5351840; doi:10.1371/journal.pone.0173358)
Supplement: S4 Table — (DOCX) [file pone.0173358.s004.docx]

**S4 Table.** Study characteristics related to inclusion and exclusion criteria per included study.

| Author, year | Description of the intervention or control group | Inclusion criteria | Exclusion criteria |
| --- | --- | --- | --- |
| Arash-Kaps 2014 [35] | Alfa | Males FD patients. | Not reported. |
| Baehner 2003 [36] | Alfa | Heterozygous females 18 years of age or older, with FD confirmed by a mutation in the a-galactosidase A gene. They should had clinical evidence of FD (with involvement of at least three organ systems). This evidence could include neuropathic pain, history of a cerebrovascular accident, left ventricular hypertrophy, or renal dysfunction consistent with FD. | Not reported. |
| Barba-Romero 2011 [37] | Alfa | Confirmed diagnostic of FD receiving ERT with agalsidase afa. | Not reported. |
| Beer 2006 [38] | Beta and natural history/untreated patients | Genetically proved FD patients. | Not reported. |
| Borgwardt 2013 [39] | Beta | Children and adolescents with FD confirmed by GLA mutation analysis. | Not reported. |
| Branton 2002 [77] | Natural history/untreated | Male FD patients with diagnosis made by the presence of the typical symptoms and signs of neuropathic pain, angiokeratoma, diarrhea and abdominal pain, proteinuria and renal failure, ocular abnormalities, or early stroke. In all patients, the clinical diagnosis was confirmed by demonstration of leukocyteα-gal A activity <15% of the value in healthy volunteers. | Not reported. |
| Collin 2011 [40] | Beta and untreated placebo patients | FD patients, either male or symptomatic females, having at least one cardiac and arterial measurement before initiation of treatment with agalsidase beta (1 mg/kg/2 weeks). | Patients treated by  algasidase alfa were excluded. |
| Choi 2008 [84] | Beta | Classical male patients and symptomatic female under ERT. | Not reported. |
| Crutchfield 1998 [78] | Natural history/untreated | Hemizygous classic FD patients with diagnosis confirmed alpha-galactosidase A assay in fibroblasts or leukocytes. | Not reported. |
| Cybulla 2013 [64] | Alfa and untreated placebo patients | FD patients. | Not reported. |
| Dehout 2004 [41] | Alfa | FD patients (9 hemizygous males, 2 heterozygous females) ease confirmed by alpha-galactosidase A assay (in males) and/or identification of a mutation in the alpha-galactosidase gene by direct sequencing. | Not reported. |
| Dominguez 2006 [93] | Beta | FD patients. | Not reported. |
| Donati 1987 [42] | Natural history/untreated | Uremic patients in renal function replacement treatment for FD. | Not reported. |
| Douglas 2015 [43] | Alfa | FD patients that participated in a phase II clinical trial of agalsidase alfa and have subsequently received ERT for up to 15 years. | Not reported. |
| Elliott 2006 [44] | Beta | Male FD patients. | Not reported. |
| Eng 2007 [65] | Natural history/untreated | Confirmed diagnosis of FD with documented plasma or leukocyte alfa-Gal A deficiency in males, or a documented alfa-Gal A deficiency and/or mutation in the alfa-Gal A gene in heterozygous females. | There was no exclusion criteria. |
| Eto 2005 [85] | Beta | Patient eligibility criteria were identical to those applicable for the phase 3 double-blind study. | Not reported. |
| Feriozzi 2012 [22] | Alfa | Adult FD patients (>18 years of age at baseline) with data on creatinine concentrations available available at baseline and after ≥5 years of ERT. | Patients not yet having completed > five years of treatment; having stage IV or V renal disease at baseline; or having missing data on renal function. |
| Fujiwara 2007 [86] | Beta | FD patients. | Not reported. |
| Gallego 2006 [45] | Alfa | FD patients confirmed by alpha-galactosidase A assay in serum and/or leukocyte, and/or mutations in the gene encoding the enzyme. | Not reported. |
| Germain 2013 [23] | Beta and natural history/untreated | Men in the untreated cohort must have had a baseline LVM measurement at the age of 18 years or older and at least one additional LVM measurement over a span of ≥2 years before the start of any agalsidase-β treatment.  Men in the treated cohort must have received agalsidase beta at an average dose at or near the recommended licensed dose of 1 mg/kg/2 weeks (≥0.9 to ≤1.1 mg/kg/2 weeks) and had a baseline LVM measurement at the age of 18 years or older and at least one posttreatment LVM measurement over a span of ≥2 years during the treatment period. | Not reported. |
| Goto 2013 [87] | Alfa | FD patients. | Not reported. |
| Guffon 2004 [46] | Beta | Patients treated who had received at least six months of treatment, and patients who participated in the phase III trial of agalsidase beta. | Not reported. |
| Guffon 2003 [47] | Natural history/untreated | Female FD patients. | Not reported. |
| Hebert 2013 [79] | Beta | Children with FD. | Not reported. |
| Hilz 2010 [66] | Beta and untreated health control patients | Hemizygous men with FD. | Not reported. |
| Hoffmann 2007 [48] | Alfa | FD patients. | Not reported. |
| Hopkin 2012 [49] | Beta | Mean FD patients who received agalsidase beta as their first source of ERT. | Not reported. |
| Hopkin 2013 [50] | Beta | Female FD patients who received agalsidase beta as their first source of ERT. | Not reported. |
| Hughes 2011 [24] | Alfa and untreated patients | FD patients over 18 years of age at entry and had received agalsidase alfa for at least 4 years. | Not reported. |
| Imbriaco 2009 [51] | Beta | Adult patients (eight men hemizygotes, three female heterozygotes) clinically assessed and diagnosed as having FD confimed by measurement of a-Gal A enzyme activity and, subsequently, analysis of the mutation in the a-Gal A gene. | Not reported. |
| Jardim 2006 [94] | Alfa | Male FD patients using agalsidase-alfa. Diagnosis of FD was confirmed by demonstration of reduced activity of α-galactosidase A in plasma or leukocytes. | Not reported. |
| Kalliokoshi 2006 [52] | Beta | FD patients. | Not reported. |
| Kampmann 2015 [25] | Alfa | FD patients receiving ERT. | Not reported. |
| Kampmann 2008 [26] | Natural history/untreated | Male and female FD patients of any age confirmed by mutation analysis. No patients had received ERT. | Patients with arterial hypertension or who were being treated with antihypertensive drugs. |
| Kim 2015 [88] | Beta | FD patients who had been on Fabrazyme for longer than 5 years. | Not reported. |
| Kisinovsky 2013 [95] | Alfa | Home infusion of agalsidase alfa in patients with FD. | Not reported. |
| Kobayashi 2008 [89] | Natural history/untreated | Japanese ERT treated patients diagnosed as classical type of FD with enzyme assay and clinical manifestations. | Not reported. |
| Kobayashi 2005 [90] | Beta | Japanese natural hisroty patients diagnosed as classical type of FD with enzyme assay and clinical manifestations. | Not reported. |
| Koskenvuo 2008 [53] | Beta | FD patients. None of the patients had a clinical history of acute myocardial infarction, were smokers or had clinical evidence or suspicion of excessive alcohol intake. | Not reported. |
| Linhart 2007 [54] | Alfa and natural history/untreated | FD patients. | Not reported. |
| Litinhorst 2004 [27] | Beta | FD patients referred to the hospital for treatment with ERT and completed at least six months of treatment. |  |
| Lubanda 2009 [55] | Beta | Male, at least 16 years, exhibited clinical manifestations consistent with FD, and levels of alfaGAL activity < 1.5 nmol/h/mL in plasma or < 4nmol/h/mg in leukocytes. | Prior ERT; creatinine 2.2; kidney transplantation; dialysis; clinically significant organic disease; or an unstable condition. |
| MacDermot 2001 [56] | Natural history/untreated | Obligate carrier FD females, over 18 years who had demographic data recorded in the registry. | Those who not provided informed consent. |
| Martins 2014 [96] | Beta | FD patients under 16 years on ERT for at least 16 months, receiving label dose. | Not reported. |
| Martins 2013 [97] | Natural history/untreated | Only natural history data (i.e., obtained from untreated patients not receiving ERT or prior to treatment with ERT) were included. | Patients for whom ERT status was unknown were excluded from the analyses to ensure that clinical events occurred prior to initiation of ERT. |
| Mehta 2009 [67] | Alfa | FD patients receiving agalsidase alfa. | Not reported. |
| Motwani 2012 [57] | Beta | FD patients aged 18 years or older with diagnosis based on measurement of enzyme activity in leucocytes and confirmed by genetic analysis in all patients. No females were pregnant and no patient was receiving ERT at the time of baseline evaluation. | Not reported. |
| Ortiz 2016 [80] | Beta | Adult FD patients aged ≥18 years at ERT initiation must have received agalsidase beta as their initial source of ERT given at or near the recommended licensed dose of 1 mg/kg every 2 weeks for up to 5 years. | Patients who developed end stage renal failure (signified by dialysis or transplant) before starting agalsidase beta were excluded because they have already had an irreversible organ failure; patients with non-classic, later-onset variant mutations. |
| Ortiz 2010 [81] | Natural history/untreated | FD patients who received renal replacement therapy (defined as either receiving chronic dialysis (≥ 40 days) or receiving a kidney transplant) during the natural history period (i.e. prior to any enzyme replacement therapy). | Not reported. |
| Pano 2014 [82] | Alfa | Treatment-naïve children with confirmed FD aged 7 years or older. | Not reported. |
| Parini 2008 [58] | Alfa | Patients with FD confirmed by enzymatic and/or molecular analysis. | Not reported. |
| Pastores 2007 [83] | Alfa | Adult male and female patients with FD and end-stage renal disease requiring dialysis (either haemodialysis or peritoneal dialysis) or who had a history of kidney transplantation were eligible for inclusion provided that their history of renal failure was consistent only with FD. In males, the diagnosis of FD was confirmed by a plasma a-Gal A level < 1.2nmol/h/ml and in females by mutation analysis. No patient had previously received ERT for FD. | Not reported. |
| Patel 2011 [68] | Natural history/untreated | Only data from untreated Fabry Registry patients or data obtained before any ERT was initiated (among patients who received treatment) were included in these analyses. | Not reported. |
| Pisani 2005 [59] | Beta | FD patients on maintenance dialysis therapy. | Not reported. |
| Ramaswami 2012 [69] | Alfa | FD patients confirmed by measurement of α-galactosidase activity in plasma, fibroblasts, or peripheral lymphocytes or leucocytes or by mutation analysis; under 18 years of age, when treatment with agalsidase alfa was initiated and who had completed at least six months of ERT. | Not reported. |
| Ries 2006 [70] | Alfa | Pediatric FD patients of both genders between the ages of 6.5 and 18 years who were naive to ERT. | Not reported. |
| Rombach 2013 [60] | Natural history/untreated | FD patients with diagnosis by enzyme-activity and DNA analysis. | Not reported. |
| Schiffmann 2014 [28] | Alfa | Pediatric FD patients (7–17 years of age at study enrollment) who had received six months of 0.2 mg/kg agalsidase alfa in study TKT023 and were within 30 (±7) days of study completion. Patients were included if they were determined to be of adequate general health, without potential safety issues or medical contraindications, and had written informed consent provided by a parent or legal guardian. | Patients were excluded if they or their legal guardian were deemed unable to understand the study requirements and potential outcomes; or if they were determined by the local investigator as unlikely to follow the study protocol. |
| Schiffmann 2009 [29] | Natural history/untreated | Diagnosed with FD during life or at the time of death, based on review of clinical findings, alfa-Gal A activities and alfa-Gal A genotypes in 96% of the patients. | Confounding renal or other diseases (e.g. diabetic nephropathy, cancer). |
| Schiffmann 2007 [30] | Alfa | Adult hemizygous male FD patients who participated in long-term agalsidase alfa clinical trials and who had demonstrated a slope of decline in estimated GFR of >5 ml/min per 1.73 m2/yr while receiving long-term treatment with agalsidase alfa at the currently recommended dosage of 0.2 mg/kg. | Not reported. |
| Schwarting 2006 [71] | Alfa | FD patients aged 20 – 60 years with a serum creatinine concentration of less than 2 mg/dl, who had been receiving ERT with agalsidase alfa for more than one month. | Not reported. |
| Sims 2009 [72] | Natural history/untreated | FD patients during the natural history period. | Not reported. |
| Sirrs 2014 [31] | Natural history/untreated | All Canadians FD patients with 18 years of age and over with confirmed diagnosis (cohort 1a, ERT and cohort 1c, naive). | Not reported. |
| Spinelli 2004 [61] | Beta | Patients with Fabry cardiac disease. | Not reported. |
| Thofehrn 2009 [98] | Alfa | FD patients with diagnosis confirmed by demonstration of reduced GALA plasma activity and mutation analysis of the GLA gene. | Not reported. |
| Tsuboi 2015a [91] | Alfa | FD naïve patients who underwent agalsidase alfa. | Not reported. |
| Tsuboi 2015b [92] | Beta | FD naïve patients who underwent agalsidase beta. | Not reported. |
| Vedder 2008 [32] | Alfa | FD patients treated with either 0.2 mg/kg agalsidase alfa or beta or 1.0 mg/kg agalsidase beta for a minimum of 12 months, and diagnosed by means of a reduced a-galactosidase A (a-Gal A) activity in leukocytes (males) and/or a mutation in the a-Gal A gene (females). | Not reported. |
| Vedder 2007 [33] | Natural history/untreated | FD patients. | Not reported. |
| Warnock 2012 [73] | Beta | FD patients aged 18 years or older who received agalsidase beta at an average dose of 1 mg/kg/2 weeks for at least two years. Patients must have had at least three serum creatinine values reported over a span of at least two years after their first agalsidase beta infusion, with at least one of the three eGFR assessments reported within three months before or after their first infusion and at least one urinary protein: creatinine ratio value reported within three months before or after their first infusion. | Not reported. |
| Watt 2010 [74] | Beta | FD patients who were treated with agalsidase beta and who had baseline and at least two yearly posttreatment health-related quality of life measurements. | Not reported. |
| Weidemann 2013 [62] | Beta and natural history/untreated | Genetically proven FD who have been treated for at least five years, and patients who were not treated with ERT because of problems with reimbursement in their countries of residence. | Not reported. |
| West 2013 [34] | Alfa | FD patients. | Not reported. |
| Whybra 2009 [63] | Alfa | Symptomatic FD women that had not been previously treated with ERT. | Not reported. |
| Wraith 2008 [75] | Beta | At enrollment, participants had to be 7-15 years of age, and at or below Tanner Stage III of pubertal development. Inclusion criteria required a clinical diagnosis of FD, documentation of reduced Gal activity for male patients and gene mutation for female patients, and at least one of the following: history of Fabry pain crises, chronic pain not effectively controlled with pain medication, or both; urine albumin level >30 mg/dL; eGFR rate <80 mL/ min; history of post-prandial abdominal pain, nausea, or vomiting; autonomic neuropathy evidenced by hypohidrosis, impaired pupillary constriction, or reduced tear production; low BMI; abbreviated P-R interval. All patients met between two and six of these criteria. | Patients who previously received ERT were not eligible. |
| Wu 2010 [76] | Natural history/untreated | For AGAL-008-00 trial: 16 years old; a current diagnosis of FD with no prior treatment with agalsidase; a clinical presentation consistent with FD; documented agal activity <.5 nmol/h/mL plasma or <4nmol/h/mg in leucocytes; and mild-to-moderate renal disease, defined as a serum creatinine (Cr) of 1.2 – 3.0 mg/dL or an estimated Cr clearance <80mL/min, if Cr was <1.2mg/dL.  For AGAL-009-00: ≥ 8 years old; a current diagnosis of FD with no prior treatment with agalsidase; and a clinical presentation consistent with FD. | Not reported. |

aGAL: lysosomal alpha-galactosidase A enzyme; BMI: body mass index; eGFR: estimated glomerular filtration rate; ERT: enzyme replacement therapy; FD: Fabry disease; Gb3: globotriao-sylceramide; LVM: left-ventricular mass; no.: number.
