# Supplementary figures and images for: Enzyme replacement therapy for Anderson-Fabry disease: A complementary overview of a Cochrane publication through a linear regression and a pooled analysis of proportions from cohort studies
Source: PLoS One. 2017 Mar 15;12(3):e0173358. doi: 10.1371/journal.pone.0173358 (PMC5351840; doi:10.1371/journal.pone.0173358)

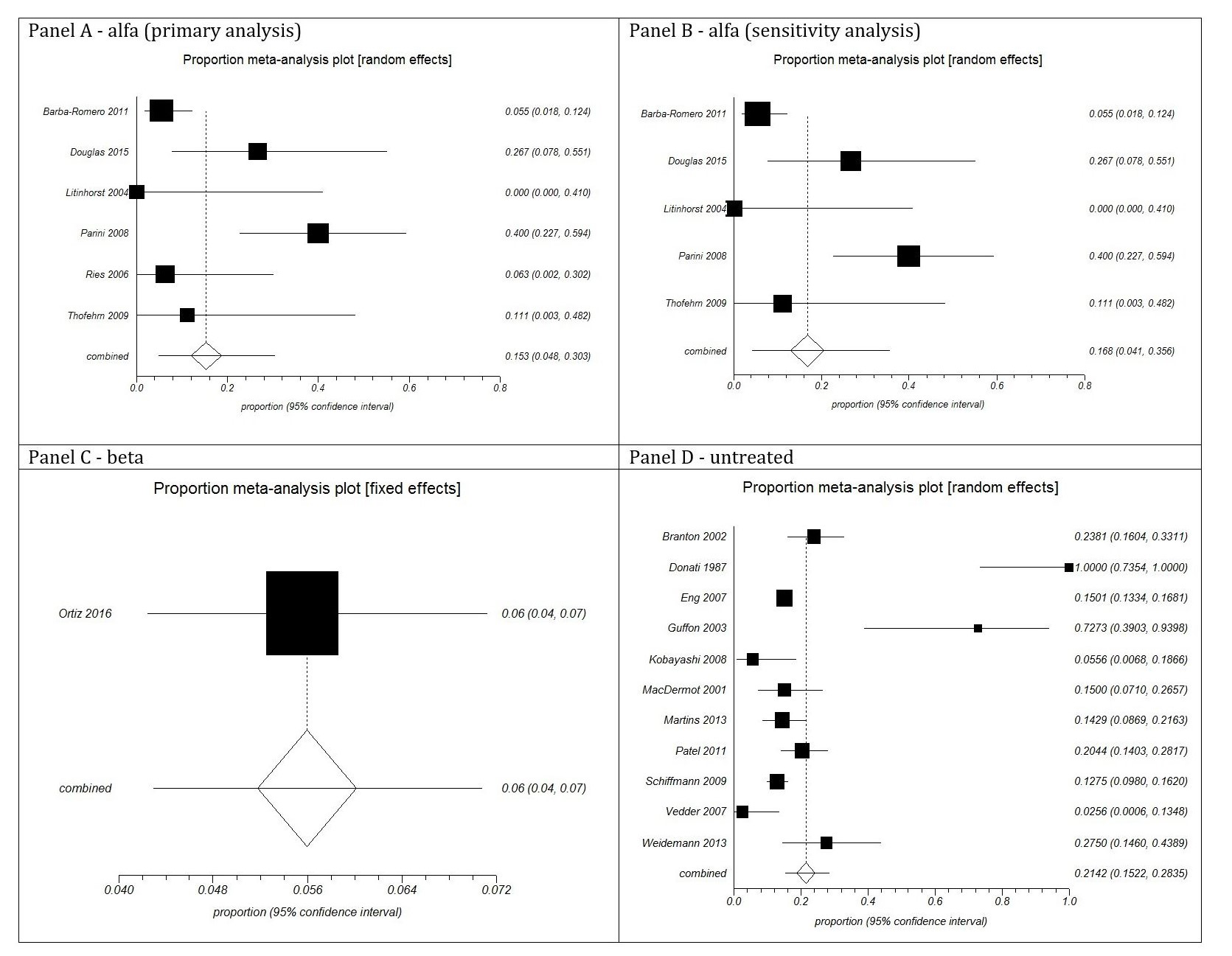

Supplement: S1 Fig — Panel A: agalsidase alfa (primary analysis). Panel B: agalsidase alfa (sensitivity analysis excluding children). Panel C: agalsidase beta. Panel D: untreated patients. (TIF) [file pone.0173358.s005.tif]

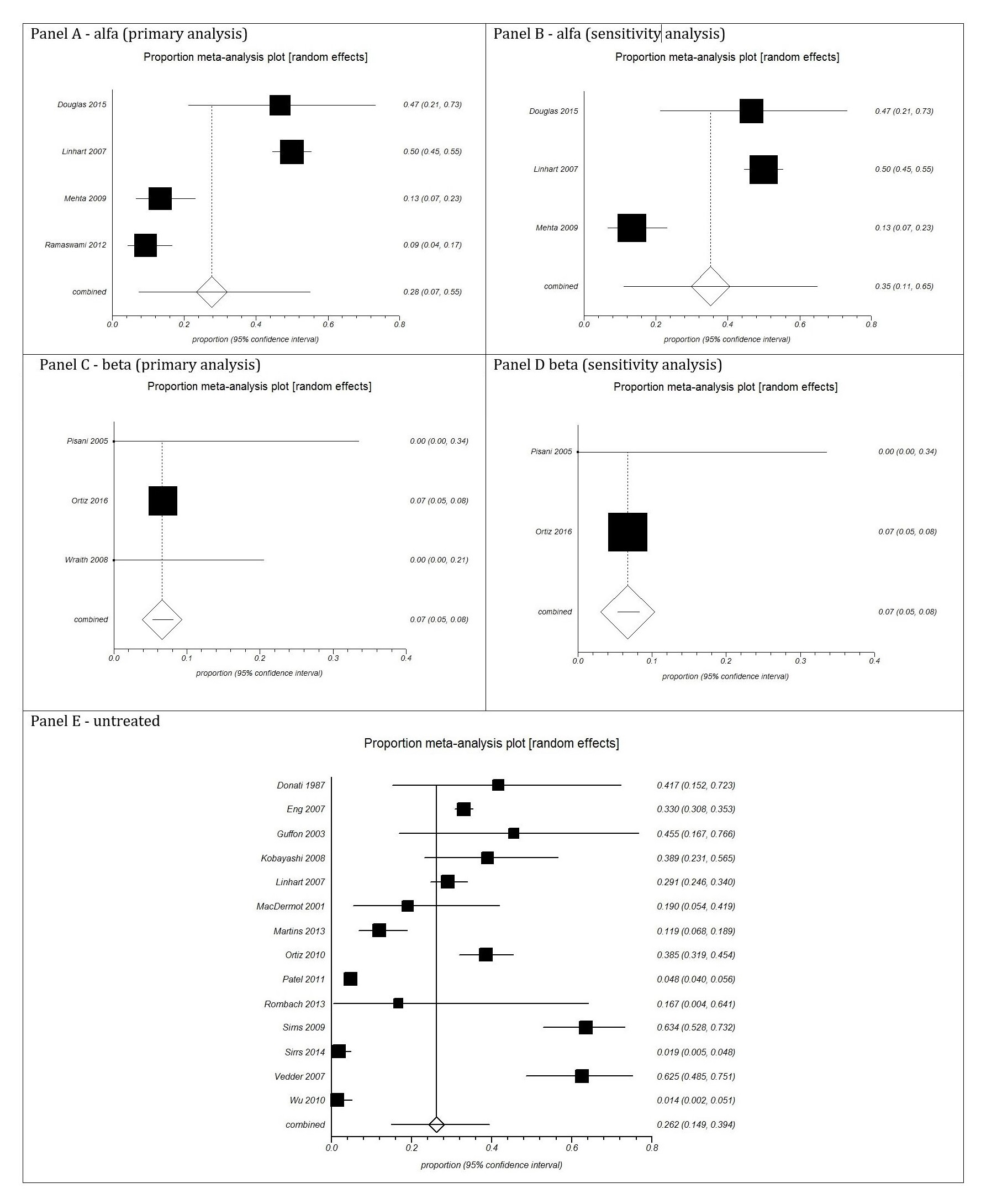

Supplement: S2 Fig — Panel A: agalsidase alfa (primary analysis). Panel B: agalsidase alfa (sensitivity analysis excluding children). Panel C: agalsidase beta (primary analysis). Panel D: agalsidase beta (sensitivity analysis excluding children). Panel E: untreated patients. (TIF) [file pone.0173358.s006.tif]

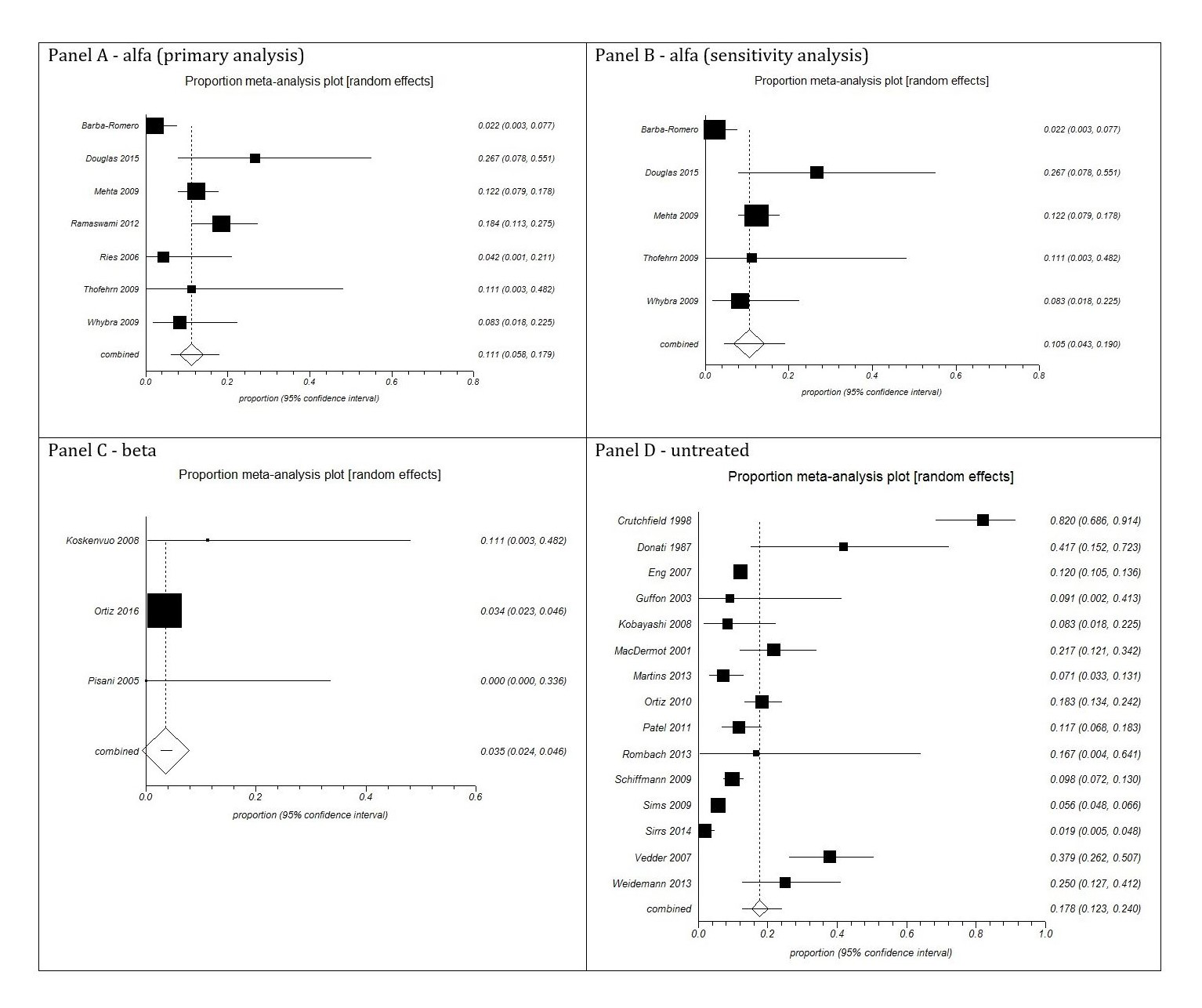

Supplement: S3 Fig — Panel A: agalsidase alfa (primary analysis). Panel B: agalsidase alfa (sensitivity analysis excluding children). Panel C: agalsidase beta. Panel D: untreated patients. (TIF) [file pone.0173358.s007.tif]

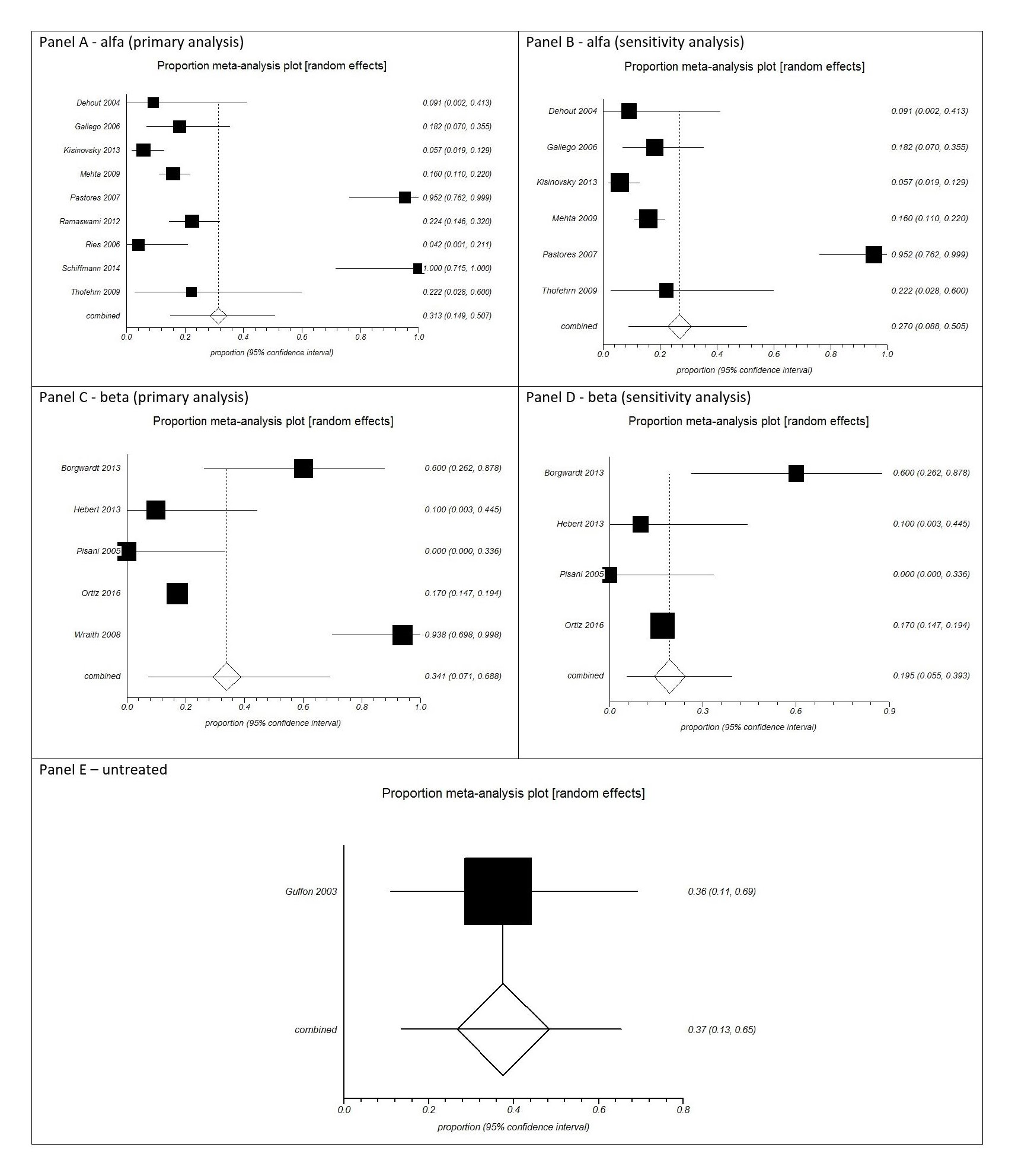

Supplement: S4 Fig — Panel A: agalsidase alfa (primary analysis). Panel B: agalsidase alfa (sensitivity analysis excluding children). Panel C: agalsidase beta (primary analysis). Panel D: agalsidase beta (sensitivity analysis excluding children). Panel E: untreated patients. (TIF) [file pone.0173358.s008.tif]

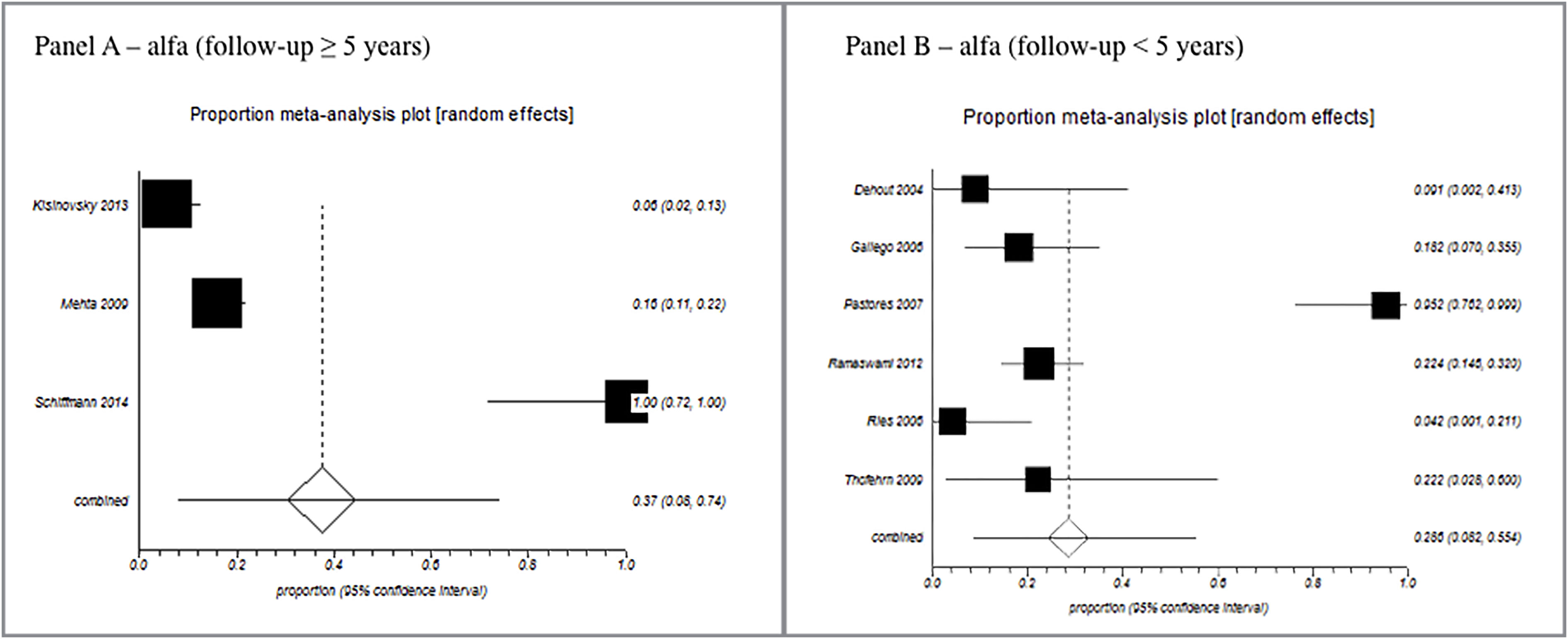

Supplement: S5 Fig — Panel A: ≥ 5 years. Panel B: < 5 years. (TIF) [file pone.0173358.s009.tif]
